# Diversity of the Swine Leukocyte Antigen Class I and II in Commercial Pig Populations

**DOI:** 10.3389/fvets.2021.637682

**Published:** 2021-04-30

**Authors:** Navapon Techakriengkrai, Teerawut Nedumpun, William T. Golde, Sanipa Suradhat

**Affiliations:** ^1^Department of Veterinary Microbiology, Faculty of Veterinary Science, Chulalongkorn University, Bangkok, Thailand; ^2^Diagnosis and Monitoring of Animal Pathogens Research Unit, Chulalongkorn University, Bangkok, Thailand; ^3^Center of Excellence in Emerging Infectious Diseases in Animals, Chulalongkorn University (CU-EIDAs), Bangkok, Thailand; ^4^Department of Vaccines and Diagnostics, Moredun Research Institute, Penicuik, United Kingdom

**Keywords:** swine leukocyte antigen, SLA, class I, class II, diversity, haplotype, commercial pigs

## Abstract

Among swine genetic markers, the highly polymorphic swine leukocyte antigen (SLA) is one of the key determinants, associated with not only immune responses but also reproductive performance and meat quality. The objective of this study was to characterize the SLA class I and II diversities in the commercial pig populations. In this study, a total number of 158 pigs (126 gilts and 32 boars) were randomly selected from different breeding herds of five major pig-producing companies, which covered ~70% of Thai swine production. The results indicate that a moderate level of SLA diversity was maintained in the Thai swine population, despite the performance-oriented breeding scheme. The highly common SLA class I alleles were SLA-1^*^08:XX, SLA-2^*^02:XX, and SLA-3^*^04:XX at a combined frequency of 30.1, 18.4, and 34.5%, respectively, whereas DRB1^*^04:XX, DQB1^*^02:XX and DQA^*^02:XX were the common class II alleles at 22.8, 33.3, and 38.6%, respectively. The haplotype Lr-32.0 (SLA-1^*^07:XX, SLA-2^*^02:XX, and SLA-3^*^04:XX) and Lr-0.23 (DRB1^*^10:XX, DQB1^*^06:XX, DQA^*^ 01:XX) was the most common SLA class I and II haplotype, at 15.5 and 14.6%, respectively. Common class I and II haplotypes were also observed, which Lr-22.15 was the most predominant at 11.1%, followed by Lr-32.12 and Lr-4.2 at 10.8 and 7.9%, respectively. To our knowledge, this is the first report of SLA class I and II diversities in the commercial pigs in Southeast Asia. The information of the common SLA allele(s) in the population could facilitate swine genetic improvement and future vaccine design.

## Introduction

The porcine major histocompatibility complex (MHC) is known as the swine leukocyte antigens (SLA) and has a very similar structure to the human leukocyte antigen (HLA). Following human, the genes of the SLA locus are among the most extensively studied MHC molecules, as reflected by the continuously expanding database (https://www.ebi.ac.uk/ipd/mhc/group/SLA). The SLA complex is located on chromosome 7 (SSC7) and consists of three gene clusters, the SLA class I, III, and II. The SLA class I and II regions encode the MHC class I and II, respectively, while the class III region encodes genes important for immune defenses and inflammation (1, 2). Due to the limited sequence homology between SLA class I and their human counterparts, the HLA class I, the SLA class I genes are designated with number, i.e., *SLA-1, 2*, and *3* to prevent misinterpretation as being direct HLA orthologs (1, 3, 4). These three SLA class I proteins are constitutively expressed and functional on the surface of all nucleated cells. On the contrary, SLA class II, which displays strong homology to HLA class II, are alphabetically designated after the HLA class II genes as *SLA-DR, DQ, DM* and *DO* (4, 5). Of the four classical SLA-II genes, 2 (DR and DQ) are constitutively expressed on the surface of professional antigen presenting cells (APC) such as dendritic cells, macrophages and B lymphocytes (1, 6, 7). Similar to other MHC loci, SLA polymorphisms reside mainly in regions encoding the peptide-binging groove, therefore SLA alleles were assigned into group according to their polymorphisms in the exon 2 and 3 for SLA class I (α_1_ and α_2_ domains) and exon 2 (β_1_ domain) for SLA class II (2).

The frequency and diversity of SLA alleles have already been characterized in several purebred populations including, Canadian Yorkshire and Landrace (8), Chinese Bama miniature pig (9), Duroc (10), German Landrace (11), Gottingen minipigs (12), Guizhou minipigs (13), Korean native pig (14), Meishan (15), Microminipig (16), Pietrain (17), Yucatan miniature pig (18), and recently, Babraham (19) and Rongshui miniature pigs (20). However, almost all of these studies were conducted in populations which were purposely bred as a resource for scientific experimentation. On the contrary, the standard crossbred pigs, produced by artificially inseminated mixed semen from purebred Duroc (D) and/or Pietrain (P) boars into 50% Yorkshire (Y) ×50% Landrace (L) crossbred sows, are the most common production pigs. These crossbred pigs are currently raised in the farming industry worldwide due to their impressive performance (fast growth rate, good feed efficiency, and carcass quality). Despite being the majority, information on SLA diversity in these crossbred populations are rather scarce, with studies conducted in the mixed US herds (50%LY/50%D and 50%L/50%Y) (21, 22), Danish herds (50%LY/50%D) (12, 23) and Belgian herds (50%L/50%P) (12). Moreover, almost all SLA class II diversity studies were in the purebred animals (8, 10, 13, 15, 18–20), with only one conducted in the US outbred population (22).

The SLA complex is one of the key determinants of swine immune responses (24–28). Additionally, some studies have reported impact of SLA on other traits including bodyweight (29), meat production (27) and reproductive traits such as fertility index, ovulation rate and litter size (28, 30, 31). Information on the commonly occurring SLA allele(s) in the population is not only important for genetic improvement but also could influence the future vaccine design. If a limited number of SLA class I and II genes dominate expression in these production pigs, it is likely they would present peptides conserved across viral strains. Even though these “T lymphocyte antigenic epitopes” would be different among SLA molecules, the viral proteome provides thousands of potential peptides and incorporating the subset of peptides that are T lymphocyte epitopes in the vaccine payload, would allow targeting of T lymphocyte responses, thereby enhancing vaccine performance (32, 33). Currently, there is no information on SLA diversity in the Thai swine population. Therefore, the objective of this study was to identify and characterize the frequencies of SLA alleles and haplotypes in the commercial pig population in Thailand.

## Materials and Methods

### Study Population

A total number of 158 (126 blood and 32 semen) samples were randomly selected from different breeding herds of the five major pig-producing companies; three from the central (A, multi-provinces, *n* = 32); (B, Ayuthaya, *n* = 24); (C, Lopburi, *n* = 35); one from the eastern (D, Chonburi, *n* = 32); and one from the western (E, Ratchaburi, *n* = 35) regions of Thailand. These five companies covered ~70% of commercial Thai swine production (2017 annual report, Department of Livestock Development, Thailand). Gilts were either 50% Yorkshire (Y) ×50% Landrace (L) (Gilts50) or 75% Yorkshire ×25% Landrace (Gilts75) crossbreeds while all boars were purebred Duroc (D). All animals were bred from farm-owned breeding stock of purebred grandparent (GP). Heparinized blood samples from gilts and semen samples from boar were collected by farm veterinarians and transported at 4°C to the laboratory within twelve hours after collection. This study has been reviewed and approved by the Faculty of Veterinary Science Animal Care and Use Committee (VET-ACUC) (protocol number 2031085).

### Low Resolution SLA Typing by Sequence-Specific Polymerase Chain Reaction (SSP-PCR)

Genomic DNA was isolated from buffy coat and semen using Qiagen DNA Blood Mini Kit (Qiagen), aliquoted and stored at −20°C until used. Low resolution SLA typing was performed by sequence-specific polymerase chain reaction (SSP-PCR) using two primer sets published previously (21, 22). These primer sets can identify 38 allelic groups of SLA class I, 15 SLA-1, 16 SLA-2, and 7 SLA-3; and 29 of SLA class II, 14 DRB1, 10 DQB1 and 5 DQA. The porcine α-actin gene (*ACTA1*) was used as internal control. PCR reaction was performed in a 10 μl reaction volume, with ~30 ng of gDNA, 5 pmol of each primer and DNA polymerase from TopTaq^TM^ Master Mix Kit (Qiagen). RNase free water was used as negative control to check for reagent contamination. The thermal cycling conditions were as follow, with initial activation at 95°C for 3 min, followed by 35 cycles of 95°C for 45 s, 64°C for 30 s and 72°C for 30 s, and a final elongation at 72°C for 10 min. PCR products were electrophoresed on a 3% agarose gels, in the presence of GeneRuler 100 bp Plus DNA Ladder (Thermo Scientific^TM^). SLA allelic groups were named according to the International Society of Animal Genetics (ISAG) and the Veterinary and Immunology Committee (VIC) of the International Union of Immunological Societies (IUIS) (4, 34) and the Immuno Polymorphism Database (IPD)-MHC SLA website (https://www.ebi.ac.uk/ipd/mhc/group/SLA). The complete typing result was summarized in Supplementary Table 1.

### Low Resolution Haplotype Allocation

Low resolution of SLA class I and II haplotypes were deduced from individual allelic groups identified in the animal and allocated according to previously published data (1, 4, 8, 10, 16, 18, 23, 35) and a SLA haplotype database kindly provided by Dr. Chak-Sum Ho (unpublished data). Unknown haplotypes were haplotypes that have never been reported elsewhere. Potential novel haplotypes were haplotypes detected in at least two animals and have not been identified elsewhere. SLA class I and II associations that were detected in at least 2 animals were identified as common SLA class I and II haplotypes.

## Results

### Diversity of SLA Class I Alleles and Haplotypes

**Figure 1:**
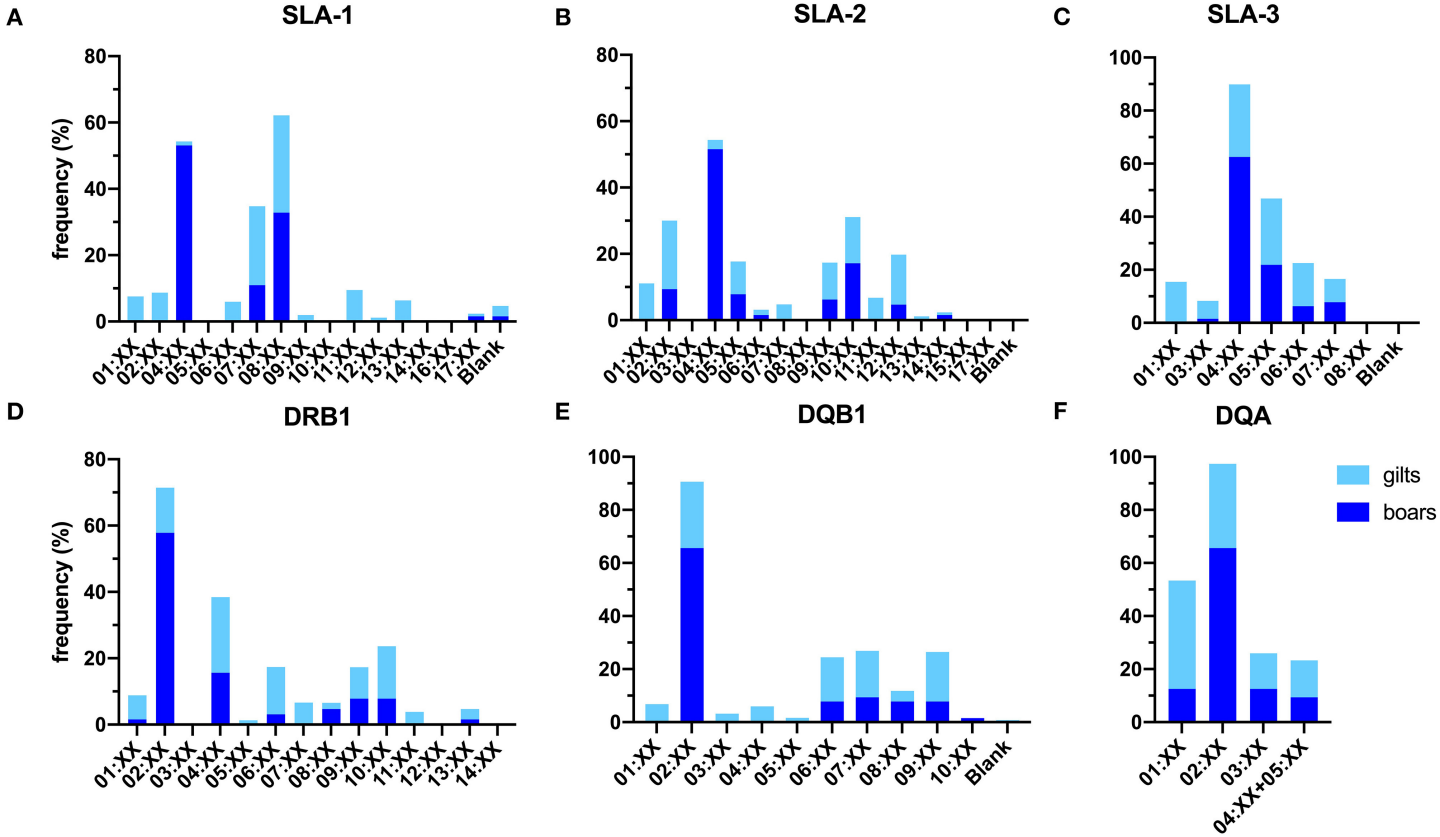
Frequencies of SLA class I **(A–C)** and class II **(D–F)** allelic groups identified in the study population. Blank indicates alleles that cannot be detected with the primer sets utilized in the current study.

Overall, a total number of 30 SLA class I allelic groups were identified in the study population, of which a higher diversity was observed in gilts than boars (24 vs. 18 from 38 SLA class I alleles). The difference in diversity was more prominent for SLA-1 where 12 in 15 groups were identified in gilts as compared to only 4 in boars (Figures 1A–C). For the SLA-1 locus, SLA-1^*^08:XX and SLA-1^*^04:XX were the most common SLA-1 allelic groups in gilts at 29.4% and boars at 53.1%, respectively (Table 1). SLA-2^*^02:XX and SLA-2^*^04:XX were the most common alleles in gilts at 20.6% and boars at 51.6%, respectively. SLA-3^*^04:XX was the most common SLA-3 allelic group in both gilts and boars at 27.4% and 62.5%, respectively. When allelic frequencies from gilts and boars were combined, SLA-1^*^08:XX, SLA-2^*^02:XX and SLA-3^*^04:XX was the most common allelic group at a frequency of 30.1, 18.4, and 34.5%, respectively.

**Table 1:** Frequency of SLA class I allelic groups.

	Allelic group*	**Boar**		**Gilt**		**Total**	
		**(2*****n*** **=** **64)**		**(2*****n*** **=** **252)**		**(2*****n*** **=** **316)**	
**SLA-1**	01:XX			19	7.5%	19	6.0%
	02:XX			22	8.7%	22	7.0%
	04:XX	34	53.1%	3	1.2%	37	11.7%
	05:XX						
	06:XX			15	6.0%	15	4.8%
	07:XX	7	10.9%	60	23.8%	67	21.2%
	08:XX	21	32.8%	74	29.4%	95	30.1%
	09:XX			5	2.0%	5	1.6%
	10:XX						
	11:XX			24	9.5%	24	7.6%
	12:XX			3	1.2%	3	1.0%
	13:XX			16	6.4%	16	5.1%
	14:XX			1	0.4%	1	0.3%
	16:XX						
	17:XX	1	1.6%	2	0.8%	3	1.0%
	Blank**	1	1.6%	8	3.2%	9	2.9%
**SLA-2**	01:XX			28	11.1%	28	8.9%
	02:XX	6	9.4%	52	20.6%	58	18.4%
	03:XX						
	04:XX	33	51.6%	7	2.8%	40	12.7%
	05:XX	5	7.8%	25	9.9%	30	9.5%
	06:XX	1	1.6%	4	1.6%	5	1.6%
	07:XX			12	4.8%	12	3.8%
	08:XX						
	09:XX	4	6.3%	28	11.1%	32	10.1%
	10:XX	11	17.2%	35	13.9%	46	14.6%
	11:XX			17	6.8%	17	5.4%
	12:XX	3	4.7%	38	15.1%	41	13.0%
	13:XX			3	1.2%	3	1.0%
	14:XX	1	1.6%	2	0.8%	3	1.0%
	15:XX						
	17:XX						
	Blank**			1	0.4%	1	0.3%
**SLA-3**	01:XX			39	15.5%	39	12.3%
	03:XX	1	1.6%	17	6.8%	18	5.7%
	04:XX	40	62.5%	69	27.4%	109	34.5%
	05:XX	14	21.9%	63	25.0%	77	24.4%
	06:XX	4	6.3%	41	16.3%	45	14.2%
	07:XX	5	7.8%	22	8.7%	27	8.5%
	08:XX						
	Blank**			1	0.4%	1	0.3%

*The study primer sets cannot identify SLA-1*03:XX, SLA-1*15:XX, SLA-2*16:XX and SLA-3*02:XX. **Blank indicates alleles that cannot be identified with the study primer sets.

There were 47 SLA class I haplotypes identified in the study population of which 28 were known haplotypes previously reported in the literature. In addition, four potential novel SLA class I haplotypes were identified following the criteria that they were detected in at least two animals (Table 2). The other 15 were reported here as unknown haplotypes as any one was detected in only a single animal. The most common SLA class I haplotype in gilts was Lr-32.0 (SLA-1^*^07:XX, SLA-2^*^02:XX, and SLA-3^*^04:XX) at 17.1%, whereas the Lr-4.0 (SLA-1^*^04:XX, SLA-2^*^04:XX, and SLA-3^*^04:XX) was the most common SLA class I haplotype in boars at 45.3%. When boars and gilts were combined, the most common haplotype was Lr-32.0 at 15.5%, followed by Lr-22.0 and Lr-4.0 at 12.7 and 9.5%, respectively. A noteworthy finding in this analysis is that, while homozygosity was rarely observed in gilts (5.6%, 7/126), almost half of the boars were homozygous (46.9%, 15/32) with Lr-4.0 being the most common homozygous haplotype at 60% (9/15) (Supplementary Table 2).

**Table 2:** Frequency of SLA class I haplotypes.

**Haplotype**	**SLA-1**	**SLA-2**	**SLA-3**	**Boar (2*****n*** **=** **64)**		**Gilt (2*****n*** **=** **252)**		**Total (2*****n*** **=** **316)**	
Lr-1.0	01:XX	01:XX	01:XX			19	7.5%	19	6.0%
Lr-2.0	02:XX	02:XX	04:XX			7	2.8%	7	2.2%
Lr-4.0	04:XX	04:XX	04:XX	29	45.3%	1	0.4%	30	9.5%
Lr-6.0	08:XX	05:XX	06:XX			1	0.4%	1	0.3%
Lr-7.0	08:XX	05:XX	07:XX	4	6.3%	13	5.2%	17	5.4%
Lr-17.0	08:XX	06:XX	03:XX			1	0.4%	1	0.3%
Lr-18.0	04:XX	06:XX	03:XX	1	1.6%			1	0.3%
Lr-21.0	07:XX	05:XX	06:XX	1	1.6%	2	0.8%	3	1.0%
Lr-22.0	08:XX	12:XX	06:XX	3	4.7%	37	14.7%	40	12.7%
Lr-25.0	11:XX	07:XX	03:XX			11	4.4%	11	3.5%
Lr-26.0	08:XX	10:XX	05:XX	10	15.6%	15	6.0%	25	7.9%
Lr-27.0	06:XX	01:XX	01:XX			2	0.8%	2	0.6%
Lr-28.0	09:XX	05:XX	07:XX			5	2.0%	5	1.6%
Lr-29.0	Blank*	09:XX	05:XX			14	5.6%	14	4.4%
Lr-32.0	07:XX	02:XX	04:XX	6	9.4%	43	17.1%	49	15.5%
Lr-35.0	13:XX	10:XX	05:XX			18	7.1%	18	5.7%
Lr-37.0	07:XX	09:XX	05:XX			11	4.4%	11	3.5%
Lr-43.0	11:XX	04:XX	04:XX			5	2.0%	5	1.6%
Lr-49.0	08:XX	04:XX	05:XX	4	6.3%	1	0.4%	5	1.6%
Lr-50.0	07:XX	12:XX	06:XX			1	0.4%	1	0.3%
Lr-57.0	02:XX	11:XX	01:XX			15	6.0%	15	4.8%
Lr-58.0	08:XX	09:XX	03:XX			1	0.4%	1	0.3%
Lr-63.0	11:XX	Blank	05:XX			1	0.4%	1	0.3%
Lr-70.0	11:XX	09:XX	03:XX			2	0.8%	2	0.6%
Lr-76.0	07:XX	11:XX	04:XX			1	0.4%	1	0.3%
Lr-80.0	08:XX	05:XX	04:XX			3	1.2%	3	1.0%
Lr-81.0	13:XX	10:XX	05:XX			1	0.4%	1	0.3%
Lr-82.0	08:XX	07:XX	03:XX			1	0.4%	1	0.3%
Potential**	17:XX	10:XX	07:XX	1	1.6%	2	0.8%	3	1.0%
Potential	04:XX	09:XX	04:XX	4	6.3%			4	1.3%
Potential	11:XX	13:XX	05:XX			2	0.8%	2	0.6%
Potential	11:XX	01:XX	01:XX			2	0.8%	2	0.6%
Unknown***	12:XX	06:XX	04:XX			1	0.4%	1	0.3%
Unknown	Blank	14:XX	04:XX	1	1.6%			1	0.3%
Unknown	04:XX	01:XX	04:XX			1	0.4%	1	0.3%
Unknown	06:XX	02:XX	04:XX			1	0.4%	1	0.3%
Unknown	04:XX	14:XX	04:XX			1	0.4%	1	0.3%
Unknown	06:XX	01:XX	07:XX			1	0.4%	1	0.3%
Unknown	07:XX	06:XX	03:XX			1	0.4%	1	0.3%
Unknown	14:XX	01:XX	07:XX			1	0.4%	1	0.3%
Unknown	Blank	02:XX	04:XX			1	0.4%	1	0.3%
Unknown	06:XX	06:XX	04:XX			1	0.4%	1	0.3%
Unknown	Blank	01:XX	04:XX			1	0.4%	1	0.3%
Unknown	Blank	13:XX	05:XX			1	0.4%	1	0.3%
Unknown	Blank	01:XX	01:XX			1	0.4%	1	0.3%
Unknown	11:XX	14:XX	Blank			1	0.4%	1	0.3%
Unknown	Blank	11:XX	04:XX			1	0.4%	1	0.3%

*Blank indicates alleles that cannot be identified with the study primer sets. **Potential indicates a possible novel haplotype that was detected in at least 2 animals. ***Unknown indicates a haplotype that was not on the current database and found in a single animal only.

### Diversity of SLA Class II Alleles and Haplotypes

Overall, 25 of the 29 SLA class II allelic groups were present in the study population. Similar to SLA class I, gilts carried more diverse SLA class II than boar, (25 vs. 19 from 29 allelic groups) (Figures 1D–F). For the DRB1, DRB1^*^04:XX and DRB1^*^02:XX were the most common DRB1 allelic groups in gilts and boars at 24.6 and 57.8%, respectively. DQB1^*^02:XX was the most common allelic group in both gilts and boars at 25.0 and 65.6%, respectively. DQA^*^01:XX and DQA^*^02:XX were the most common allelic groups in gilts at 40.9% and boars at 65.6% (Table 3). When allelic frequencies from gilts and boars were combined, DRB1^*^04:XX, DQB1^*^02:XX, and DQA^*^02:XX were the most common allelic groups at a combined frequency of 22.8, 33.2, and 38.6%, respectively.

**Table 3:** Frequency of SLA class II allelic groups.

	**Allelic group**	**Boar (2*****n*** **=** **64)**		**Gilt (2*****n*** **=** **252)**		**Total (2*****n*** **=** **316)**	
**DRB1**	01:XX	1	1.6%	22	8.7%	23	7.3%
	02:XX	37	57.8%	6	2.4%	43	13.6%
	03:XX						
	04:XX	10	15.6%	62	24.6%	72	22.8%
	05:XX			4	1.6%	4	1.3%
	06:XX	2	3.1%	43	17.1%	45	14.2%
	07:XX			21	6.7%	21	6.7%
	08:XX	3	4.7%	3	1.2%	6	1.9%
	09:XX	5	7.8%	25	9.9%	30	9.5%
	10:XX	5	7.8%	45	17.9%	50	15.8%
	11:XX			12	4.8%	12	3.8%
	12:XX						
	13:XX	1	1.6%	9	3.6%	10	3.2%
	14:XX						
**DQB1**	01:XX			17	6.8%	17	5.4%
	02:XX	42	65.6%	63	25.0%	105	33.2%
	03:XX			8	3.2%	8	2.5%
	04:XX			15	6.0%	15	4.8%
	05:XX			4	1.6%	4	1.3%
	06:XX	5	7.8%	42	16.7%	47	14.9%
	07:XX	6	9.4%	44	17.5%	50	15.8%
	08:XX	5	7.8%	10	4.0%	15	4.8%
	09:XX	5	7.8%	47	18.7%	52	16.5%
	10:XX	1	1.6%			1	0.3%
	Blank*			2	0.8%	2	0.6%
**DQA**	01:XX	8	12.5%	103	40.9%	111	35.1%
	02:XX	42	65.6%	80	31.8%	122	38.6%
	03:XX	8	12.5%	34	13.5%	42	13.3%
	04:XX+ 05:XX	6	9.4%	35	13.9%	41	13.0%

*Blank indicates alleles that cannot be identified with the study primer sets.

There were 26 SLA class II haplotypes identified in this study including 21 known, 4 unknown and 1 potential novel haplotype (Table 4). Lr-0.23 (DRB1^*^10:XX, DQB1^*^06:XX, and DQA^*^01:XX) was the most common haplotype in gilts at 16.3% and Lr-0.2 (DRB1^*^02:XX, DQB1^*^02:XX, and DQA^*^02:XX) was the most common in boars at 57.8%. When boars and gilts were combined, the most common haplotype was Lr-0.23 at 14.6%, followed by Lr-0.12 at 13.6% and Lr-0.2 at 13.3%. Interestingly, homozygosity was even more prominent with respect to SLA class II where 7.1% of gilts (9/126) and 71.9% of boars (23/32) were typed as homozygous. The most common homozygous haplotype was Lr-0.2 at 50% (16/32) (Supplementary Table 2).

**Table 4:** Frequency of SLA class II haplotypes.

**Haplotype**	**DRB1**	**DQB1**	**DQA**	**Boar (2*****n*** **=** **64)**		**Gilt (2*****n*** **=** **252)**		**Total (2*****n*** **=** **316)**	
Lr-0.1	01:XX	01:XX	01:XX			17	6.8%	17	5.4%
Lr-0.2	02:XX	02:XX	02:XX	37	57.8%	5	2.0%	42	13.3%
Lr-0.4	02:XX	04:XX	02:XX			1	0.4%	1	0.3%
Lr-0.5	05:XX	02:XX	02:XX	1	1.6%			1	0.3%
Lr-0.6	05:XX	08:XX	01:XX			4	1.6%	4	1.3%
Lr-0.8	08:XX	02:XX	02:XX			3	1.2%	3	1.0%
Lr-0.10	04:XX	08:XX	03:XX	4	6.3%	4	1.6%	8	2.5%
Lr-0.11	09:XX	04:XX	03:XX			2	0.8%	2	0.6%
Lr-0.12	06:XX	07:XX	01:XX	3	4.7%	40	15.9%	43	13.6%
Lr-0.13	04:XX	03:XX	02:XX			1	0.4%	1	0.3%
Lr-0.14	09:XX	08:XX	03:XX	1	1.6%	2	0.8%	3	1.0%
Lr-0.15	04:XX	02:XX	02:XX	3	4.7%	35	13.9%	38	12.0%
Lr-0.19	04:XX	07:XX	03:XX			21	8.3%	24	7.6%
Lr-0.21	01:XX	05:XX	04:XX+05:XX			4	1.6%	4	1.3%
Lr-0.22	06:XX	02:XX	02:XX	1	1.6%	2	0.8%	3	1.0%
Lr-0.23	10:XX	06:XX	01:XX	5	7.8%	41	16.3%	46	14.6%
Lr-0.24	07:XX	02:XX	02:XX			21	8.3%	21	6.7%
Lr-0.25	13:XX	09:XX	04:XX+05:XX	1	1.6%	9	3.6%	10	3.2%
Lr-0.26	11:XX	04:XX	02:XX			12	4.8%	12	3.8%
Lr-0.27	09:XX	09:XX	04:XX+05:XX	4	6.3%	20	7.9%	24	7.6%
Lr-0.36	01:XX	10:XX	04:XX	1	1.6%			1	0.3%
Potential**	10:XX	02:XX	03:XX			4	1.6%	4	1.3%
Unknown***	01:XX	Blank*	04:XX+05:XX			1	0.4%	1	0.3%
Unknown	06:XX	02:XX	04:XX			1	0.4%	1	0.3%
Unknown	04:XX	06:XX	01:XX			1	0.4%	1	0.3%
Unknown	09:XX	09:XX	03:XX			1	0.4%	1	0.3%

*Blank indicates alleles that cannot be identified with the study primer sets. **Potential indicates a possible novel haplotype that was detected in at least 2 animals. ***Unknown indicates a haplotype that was not on the current database and found in a single animal only.

### Commonly Occurring SLA Class I and Class II Haplotypes

When SLA class I and class II haplotypes were analyzed together, it was apparent that some class I haplotypes were presented with certain class II haplotypes and were then identified as commonly occurring SLA class I and class II haplotypes. From the 32 SLA class I (28 known + 4 potential) and 22 class II (21 known + 1 potential), 33 commonly occurring haplotypes were identified in the study population (Table 5). The most common haplotypes observed in boars was Lr-4.2 at 37.5% whereas Lr-22.15 and Lr-32.12 were in gilts, both at 12.7%. Interestingly, we observed a relatively high frequency of animals that were homozygous in all 6 SLA loci (10.8%, 17/158) of which 14 were boars and 3 were gilts (Supplementary Table 2). In boars, Lr-4.2 was the most common homozygous haplotype at 50% (7/14 boars), followed by Lr-7.23 at 14.3% (2/14 boars), whereas Lr-22.15 was most common in gilts (2/3).

**Table 5:** Common SLA haplotypes^*^.

**Haplotype**	**Boar (2*****n*** **=** **64)**		**Gilt (2*****n*** **=** **252)**		**Total (2*****n*** **=** **316)**	
Lr-1.1			13	5.2%	13	4.1%
Lr-1.23			2	0.8%	2	0.6%
Lr-2.19			4	1.6%	4	1.3%
Lr-2.23			2	0.8%	2	0.6%
Lr-4.2	24	37.5%	1	0.4%	25	7.9%
Lr-4.10	2	3.1%			2	0.6%
Lr-4.19	2	3.1%			2	0.6%
Lr-7.23	4	6.3%	12	4.8%	16	5.1%
Lr-21.1			2	0.8%	2	0.6%
Lr-22.15	3	4.7%	32	12.7%	35	11.1%
Lr-25.25			9	3.6%	9	2.9%
Lr-26.2	6	9.4%	1	0.4%	7	2.2%
Lr-26.10	1	1.6%	1	0.4%	2	0.6%
Lr-26.23	1	1.6%	8	3.2%	9	2.9%
Lr-28.8			2	0.8%	2	0.6%
Lr-28.23			3	1.2%	3	1.0%
Lr-29.24			11	4.4%	11	3.5%
Lr-32.2	3	4.7%	1	0.4%	4	1.3%
Lr-32.12	2	3.1%	32	12.7%	34	10.8%
Lr-32.19	1	1.6%	7	2.8%	8	2.5%
Lr-35.10			2	0.8%	2	0.6%
Lr-35.12			2	0.8%	2	0.6%
Lr-35.23			12	4.8%	12	3.8%
Lr-37.24			3	1.2%	3	1.0%
Lr-37.27			6	2.4%	6	1.9%
Lr-43.27			4	1.6%	4	1.3%
Lr-49.2	3	4.7%			3	1.0%
Lr-57.24			2	0.8%	2	0.6%
Lr-57.26			12	4.8%	12	3.8%
Lr-70.27			2	0.8%	2	0.6%
Lr-80.21			2	0.8%	2	0.6%
Potential 1**+ Lr-0.11			2	0.8%	2	0.6%
Potential 2	3	4.7%			3	1.0%
+ Lr-0.27						

*Only SLA class I and II haplotypes detected in at least two animals were shown. Potential SLA class I haplotype indicates a possible novel haplotype that was detected in at least two animals. **Potential 1 was SLA-1*17:XX, SLA-2*10:XX, SLA-3*07:XX and potential 2 was SLA-1*04:XX, SLA-2*09:XX, SLA-3*04:XX.

## Discussion

In this study, SLA class I and II diversities of the breeding parent stocks from 5 major pig producing companies in Thailand were determined. According to the 2017 annual report of the Department of Livestock Development of Thailand, these five companies accounted for ~70% of Thai swine production. The purebred Duroc boars and Yorkshire/Landrace crossbred gilts were produced from company owned purebred great-grandparent (GGP) and grandparent (GP) stocks, which have been kept and improved regularly by their breeders. Although some degree of genetic similarities might be present as the GGP stocks were originally imported from the same European or United States (US) breeding companies, they are considered genetically unique. Piglets from these parent stocks are either raised in the company farms or sold to small-and-medium size farms as fattening pigs. The gilts themselves and semen from boars with desirable traits are often sold to medium size farms as well. Therefore, studying these breeding stocks provides a good estimation of SLA diversity in Thai commercial pigs.

Regarding SLA class I diversity, Thai pigs appeared to be relatively more diverse than other outbred pig populations, with 30/38 allelic groups present compared to 24/38 in both the Danish (23) and the US (21) studies using the same SSP-PCR method. Further, 17/38 allelic groups were present in mixed population of Belgian, Danish and Kenyan fattening pigs by next-generation sequencing (12). The higher diversity observed in the Thai population was mainly due to the SLA-1 locus in which 12/15 allelic groups were detected as compared to 7 in the Danish (23) and 8 in the US (21) and 8 in the mixed fattening herds (12).

As for the SLA class II, a study conducted in the US outbred population reported similar level of diversity in which 25 of 29 allelic groups were detected (22). A slightly narrower diversity at 20/29 allelic groups was observed in German Landrace (11) and 7/24 and 10/24 allelic groups (DRB1 and DQB1, only) in Canadian Landrace and Yorkshire, respectively (8). The higher diversity observed in Thai population was not due to the enrollment of both Duroc boars and Yorkshire/Landrace gilts, as all but one of the alleles detected in boars (DQB1^*^10:XX in single boar) were present in gilts. Thus, the finding reflects the actual SLA class II diversity of the study population (Figures 1D–F, Table 3).

In general, common SLA-I alleles observed in the Thai swine population is similar to the European outbred swine population in which SLA-1^*^08:XX, SLA-2^*^02:XX, and SLA-3^*^04:XX were also the most common in the Danish outbred herd (23) (Supplementary Table 3). Although, another study in Danish outbred pigs reported a predominance of SLA-1^*^04:XX, SLA-2^*^04:XX, and SLA-3^*^04:XX. The number of samples in that study was rather limited, with only 13 animals from a single farm, and might not reflect the true diversity of the whole Danish population (12). SLA-1^*^08:XX and SLA-3^*^04:XX were also the most common alleles in the purebred German Landrace (11). In comparison, the most common SLA class I alleles in the three-breed-cross US pig population (PCV+KSU) were SLA-1^*^04:XX, SLA-2^*^04:XX and SLA-3^*^04:XX, with SLA-1^*^08:XX being the fourth (Supplementary Table 3) (21).

A larger number of SLA class I haplotypes was also observed in the current study, with 32 (28 known + 4 potential) haplotypes identified, compared to 19 in the Danish (23) and 23 in the US study (21) (Supplementary Table 4). However, the dominant haplotype was different among the three populations, with Lr-4.0 being the most common haplotype in both the Danish and US cohort whereas it was Lr-32.0 in the Thai pig population (12, 21, 23). Lr-32.0 was the second most common in the Danish pigs whereas it was almost absent in the US study (21, 23). As Duroc boars and Yorkshire/Landrace gilts were typed separately in this study, it was apparent that Lr-32.0 was more common in gilts while Lr-4.0 was common in boars. In fact, Lr-4.0 was almost exclusively present in boars (20/32 boars vs. 1/126 gilts). Lr-22.0, the second most common haplotype, was also more common in Thai gilts. A similar result was reported from the US cohort where Lr-22.0 was more common in Yorkshire/Landrace crossbreeds (Big Pig,) than three-breed-cross pigs (KSU+PCV) (21) (Supplementary Table 4). Altogether, it appears that in the Thai swine population, Lr-4.0 mainly comes from the Duroc line while the other common haplotypes such as Lr-32.0 and Lr-22.0 are from Yorkshire/Landrace crossbred gilts. Lr-4.0 is generally considered the most common SLA class I haplotype in commercial pigs worldwide (12, 21, 23). Noteworthily, Lr-4.0 was present at a limited frequency in population without Duroc such as the German Landrace (11) or even absent in Austrian Pietrain (17). A recent study reporting high frequency of SLA-1^*^04:XX (Lr-4.0) in Duroc and SLA-1^*^08:XX (Lr-22.0) in purebred Yorkshire and Landrace, also supports this observation (36).

The common SLA class II alleles, DRB1^*^04:XX, DQB1^*^02:XX and DQA^*^02:XX observed in this study was similar to the US study (37) (Supplementary Table 5). In comparison with the German Landrace, where DRB1^*^06:XX was the most common allele (11), DRB1^*^06:XX ranked the third in the Thai and the fourth in the US swine populations, respectively. The second most common allele in the Thai population, DRB1^*^10:XX, was the third and the fifth in the German Landrace and US, respectively. The observed larger frequency of DRB1^*^10:XX in Thai gilts was in agreement with its exclusive presence in the US Yorkshire/Landrace crossbreeds (Big Pig). The high frequencies of DRB1^*^02:XX and DRB1^*^04:XX in the Thai pigs explains the abundance of DQB1^*^02:XX and DQA^*^02:XX, as they both belonged to the Lr-0.2 and Lr-0.15 haplotypes, the third and fourth common class II haplotype (Tables 3, 4). Similarly, low frequencies of these two alleles correlates to the absence of these haplotypes in the German and Canadian Landrace (8, 11). The higher frequencies of Lr-0.23 and Lr-0.12 observed in Thai gilts were in agreement with the German, Canadian Landrace and Yorkshire populations (Supplementary Table 6). Further, both haplotypes were rather low in both the US Yorkshire/Landrace and three-breed-cross cohorts (22).

As there were limited number of studies investigating both the SLA I and II diversity in outbred pig populations, it was difficult to compare the common haplotypes observed in this study (Supplementary Table 7). Nevertheless, one of the predominant haplotypes Lr-32.12 in Thai gilts has been previously reported in both the Canadian and German Landrace (8, 11). However, the equally prevalent haplotype Lr-22.15 was not detected in those studies as both the Canadian and German Landrace population lack Lr-22.0. Other common haplotypes such as Lr-7.23 and Lr-26.23 were also reported in the Canadian Landrace and Yorkshire. While Lr-28.23 was observed at a high frequency of 13.6% in the Canadian Landrace, it was low in both this study at 1.0% and the German Landrace at 0.8%. The dominant Lr-4.2 haplotype observed in Thai Duroc boars was not found in the study conducted in a selected Japanese Duroc line (10). The absence of this haplotype in the Japanese study suggests a highly unique genetic background of the population, as this haplotype is commonly present in swine breeds worldwide (8, 12, 21, 23, 36). Therefore, further study conducted in a larger population of Duroc from different sources is required to confirm the presence of Lr-4.2 haplotype reported in this study.

In this study, while most gilts were heterozygous (88.9%, 112/126), almost all boars were homozygous (84.4% 27/32). The high proportion of homozygotes observed in boars could possibly be due to the smaller number of Duroc GGP stocks in comparison with Yorkshire and Landrace in most farms. In Farm D where the largest number of homozygotes were observed (3/22 gilts and 9/10 boars), there was only 30 purebred GGP Duroc sows. Another possibility was sampling bias toward siblings of the same parents. Unfortunately, we were unable to address this possibility as semen samples were submitted to the laboratory without pedigree.

Lacking pedigree also limited the validation of potential and unknown haplotypes observed in this study. As these haplotypes have never been reported elsewhere, they could possibly be new haplotypes. The probability of being a true new haplotype would be higher for the five potential haplotypes as they were detected in more than one pig. However, its novelty requires confirmation either by typing their parents and offspring, or sequencing-based technique.

Although the SSP-PCR method used in this study has been performed successfully in several inbred and outbred pigs worldwide (11, 15, 17, 21, 22, 37), results are limited to the known alleles specific to the primer sets. Due to the ever-expanding SLA database, the primer sets used in this study can identify only 79.8% (67/84) of the allelic groups on the current database and do not detect the new 12 groups of the SLA class I (7 SLA-1and 5 SLA-2) and, 5 groups of the SLA class II (3 DRB1, 1 DQB1, and 1 DQA). This limitation might explain blank PCRs observed in this study, especially on SLA-1, similar to the German Landrace and US Big pig studies (11, 21). However, missing these new 17 groups does not undermine the utility of SSP-PCR technique as these new groups are not as diverse as the 67 groups identifiable by the current primer sets. At the allele level, the current primer sets missed only 7.8% (32/407) of the alleles available on the database. In addition, as those new allelic groups were mostly found in purebred resource breeds, such as SLA-1^*^19:03 and SLA-2^*^20:01 in Yucatan miniature pig (18) or indigenous breeds such as SLA-2^*^16:02 in Korean native pig (14), presence of these alleles should be low in the Thai commercial pigs. Further investigation using sequencing-based technique is required to characterize these blank PCRs and address the possibility of unknown introduction of Thai indigenous breeds such as Ka Done and Puang (38).

The current pig breeding scheme in Thailand focuses mainly on performance and productivity traits such as carcass quality and percentage of lean meat on the sire line, and number of piglets per litter on the sow line (38, 39). This scheme can lead to the narrowing of SLA diversity due to inbreeding and, in turn, increased population vulnerability to novel pathogens that often emerge in industrialized farming practice, including pig farming (40, 41). In this study, the results indicate that a moderate level of SLA diversity was maintained in the Thai swine population despite the performance-oriented breeding scheme. It might be due to the importation of both European and US genetic resources and different breeding practice conducted by each company. This speculation was supported by differences in both the common alleles and allelic frequencies among the five companies (Supplementary Table 1).

To our knowledge, this study is the first report on SLA diversity in the commercial pig population in Thailand and Southeast Asia. The SLA haplotypes observed in Thai pig populations are shared by several other populations (Supplementary Table 8). This information will facilitate genetic improvement by selective breeding and the establishment of genetically controlled animal model for further studies. Also, it will facilitate the identification of candidate antigens stimulating T lymphocytes by focusing on conserved antigenic epitopes shared by diverse strains of a pathogenic virus and presented by the more common SLA haplotypes expressed in production pigs.

## Data Availability Statement

The raw data supporting the conclusions of this article will be made available by the authors, without undue reservation.

## Ethics Statement

The animal study was reviewed and approved by The Faculty of Veterinary Science Animal Care and Use Committee (VET-ACUC) (protocol number 2031085).

## Author Contributions

NT and SS designed the study. NT performed the study and analyzed the data. NT, TN, WG, and SS prepared the manuscript. All authors read and approved the final manuscript.

## Conflict of Interest

The authors declare that the research was conducted in the absence of any commercial or financial relationships that could be construed as a potential conflict of interest.
