# Supplementary material for: Diversity of the Swine Leukocyte Antigen Class I and II in Commercial Pig Populations
Source: Front Vet Sci. 2021 Apr 30;8:637682. doi: 10.3389/fvets.2021.637682 (PMC8121083; doi:10.3389/fvets.2021.637682)
Supplement: Supplementary file 1 [file Data_Sheet_1.pdf]

Supplementary Table 1 Low resolution SLA class I and II typing and haplotypes of the 5 Thai major breeding herds

All gilts were 50% Yorkshire x 50% Landrace apart from Farm A gilt75, which were 75% Yorkshire x 25% Landrace crossbreds. All the boars were purebred Duroc. Unknown haplotypes were haplotypes that have never been reported elsewhere. Potential novel haplotypes were haplotypes detected in at least 2 animals and have not been identified elsewhere.

1. Farm A (n = 32)

1.1 Gilt50 (n = 13)

	SLA-1	SLA-2	SLA-3	Haplotype	DRB1	DQB1	DQA	Haplotype
AG5001	07:XX	02:XX	04:XX	Lr-32.0	06:XX	07:XX	01:XX	Lr-0.12
	08:XX	12:XX	06:XX	Lr-22.0	04:XX	07:XX	03:XX	Lr-0.19
AG5002	07:XX	02:XX	04:XX	Lr-32.0	06:XX	07:XX	01:XX	Lr-0.12
	07:XX	09:XX	05:XX	Lr-37.0	06:XX	02:XX	02:XX	Lr-0.22
AG5003	07:XX	02:XX	04:XX	Lr-32.0	06:XX	07:XX	01:XX	Lr-0.12
	08:XX	12:XX	06:XX	Lr-22.0	04:XX	02:XX	02:XX	Lr-0.15
AG5004	07:XX	02:XX	04:XX	Lr-32.0	06:XX	07:XX	01:XX	Lr-0.12
	08:XX	12:XX	06:XX	Lr-22.0	04:XX	02:XX	02:XX	Lr-0.15
AG5005	07:XX	02:XX	04:XX	Lr-32.0	06:XX	07:XX	01:XX	Lr-0.12
	02:XX	02:XX	04:XX	Lr-2.0	04:XX	07:XX	03:XX	Lr-0.19
AG5006	07:XX	02:XX	04:XX	Lr-32.0	06:XX	07:XX	01:XX	Lr-0.12
	07:XX	12:XX	06:XX	Lr-50.0	04:XX	07:XX	03:XX	Lr-0.19
AG5007	07:XX	02:XX	04:XX	Lr-32.0	06:XX	07:XX	01:XX	Lr-0.12
	08:XX	12:XX	06:XX	Lr-22.0	04:XX	02:XX	02:XX	Lr-0.15
AG5008	07:XX	02:XX	04:XX	Lr-32.0	06:XX	07:XX	01:XX	Lr-0.12
	02:XX	02:XX	04:XX	Lr-2.0	04:XX	07:XX	03:XX	Lr-0.19
AG5009	07:XX	02:XX	04:XX	Lr-32.0	06:XX	07:XX	01:XX	Lr-0.12
	08:XX	12:XX	06:XX	Lr-22.0	04:XX	02:XX	02:XX	Lr-0.15
AG5010	07:XX	02:XX	04:XX	Lr-32.0	06:XX	07:XX	01:XX	Lr-0.12
	08:XX	12:XX	06:XX	Lr-22.0	04:XX	02:XX	02:XX	Lr-0.15
AG5011	07:XX	02:XX	04:XX	Lr-32.0	06:XX	07:XX	01:XX	Lr-0.12
	08:XX	12:XX	06:XX	Lr-22.0	04:XX	02:XX	02:XX	Lr-0.15
AG5012	07:XX	02:XX	04:XX	Lr-32.0	06:XX	07:XX	01:XX	Lr-0.12
	07:XX	09:XX	05:XX	Lr-37.0	07:XX	02:XX	02:XX	Lr-0.24
AG5013	07:XX	02:XX	04:XX	Lr-32.0	06:XX	07:XX	01:XX	Lr-0.12
	08:XX	12:XX	06:XX	Lr-22.0	04:XX	02:XX	02:XX	Lr-0.15

1.2 Gilt75 (n = 13)

	SLA-1	SLA-2	SLA-3	Haplotype	DRB1	DQB1	DQA	Haplotype
AG7501	08:XX	12:XX	06:XX	Lr-22.0	04:XX	02:XX	02:XX	Lr-0.15
	17:XX	10:XX	07:XX	Potential	09:XX	04:XX	03:XX	Lr-0.11
AG7502	08:XX	12:XX	06:XX	Lr-22.0	04:XX	02:XX	02:XX	Lr-0.15
	01:XX	01:XX	01:XX	Lr-1.0	01:XX	01:XX	01:XX	Lr-0.1
AG7503	07:XX	02:XX	04:XX	Lr-32.0	06:XX	07:XX	01:XX	Lr-0.12
	02:XX	02:XX	04:XX	Lr-2.0	04:XX	07:XX	03:XX	Lr-0.19
AG7504	08:XX	12:XX	06:XX	Lr-22.0	04:XX	02:XX	02:XX	Lr-0.15
	01:XX	01:XX	01:XX	Lr-1.0	01:XX	01:XX	01:XX	Lr-0.1

	SLA-1	SLA-2	SLA-3	Haplotype	DRB1	DQB1	DQA	Haplotype
AG7505	07:XX	02:XX	04:XX	Lr-32.0	06:XX	07:XX	01:XX	Lr-0.12
	08:XX	12:XX	06:XX	Lr-22.0	04:XX	02:XX	02:XX	Lr-0.15
AG7506	07:XX	02:XX	04:XX	Lr-32.0	06:XX	07:XX	01:XX	Lr-0.12
	04:XX	04:XX	04:XX	Lr-4.0	02:XX	02:XX	02:XX	Lr-0.2
AG7507	01:XX	01:XX	01:XX	Lr-1.0	01:XX	01:XX	01:XX	Lr-0.1
	08:XX	05:XX	07:XX	Lr-7.0	10:XX	06:XX	01:XX	Lr-0.23
AG7508	07:XX	02:XX	04:XX	Lr-32.0	06:XX	07:XX	01:XX	Lr-0.12
	11:XX	07:XX	03:XX	Lr-25.0	13:XX	09:XX	04:XX	Lr-0.25
AG7509	07:XX	02:XX	04:XX	Lr-32.0	06:XX	07:XX	01:XX	Lr-0.12
	08:XX	12:XX	06:XX	Lr-22.0	04:XX	02:XX	02:XX	Lr-0.15
AG7510	08:XX	05:XX	07:XX	Lr-7.0	10:XX	06:XX	01:XX	Lr-0.23
	08:XX	06:XX	03:XX	Lr-17.0	06:XX	07:XX	01:XX	Lr-0.12
AG7511	01:XX	01:XX	01:XX	Lr-1.0	01:XX	01:XX	01:XX	Lr-0.1
	17:XX	10:XX	07:XX	Potential	09:XX	04:XX	03:XX	Lr-0.11
AG7512	11:XX	07:XX	03:XX	Lr-25.0	06:XX	07:XX	01:XX	Lr-0.12
	11:XX	07:XX	03:XX	Lr-25.0	13:XX	09:XX	04:XX+ 05:XX	Lr-0.25
AG7513	08:XX	12:XX	06:XX	Lr-22.0	04:XX	02:XX	02:XX	Lr-0.15
	12:XX	06:XX	004:XX	Unknown	01:XX	Blank	04:XX+ 05:XX	Unknown

1.3 Boar (n = 6)

	SLA-1	SLA-2	SLA-3	Haplotype	DRB1	DQB1	DQA	Haplotype
AB01	04:XX	04:XX	04:XX	Lr-4.0	02:XX	02:XX	02:XX	Lr-0.2
	04:XX	04:XX	04:XX	Lr-4.0	02:XX	02:XX	02:XX	Lr-0.2
AB02	07:XX	02:XX	04:XX	Lr-32.0	06:XX	07:XX	01:XX	Lr-0.12
	07:XX	02:XX	04:XX	Lr-32.0	06:XX	07:XX	01:XX	Lr-0.12
AB03	07:XX	02:XX	04:XX	Lr-32.0	04:XX	02:XX	02:XX	Lr-0.15
	08:XX	12:XX	06:XX	Lr-22.0	04:XX	07:XX	03:XX	Lr-0.19
AB04	04:XX	04:XX	04:XX	Lr-4.0	02:XX	02:XX	02:XX	Lr-0.2
	17:XX	10:XX	07:XX	Potential	09:XX	08:XX	03:XX	Lr-0.14
AB05	08:XX	12:XX	06:XX	Lr-22.0	04:XX	02:XX	02:XX	Lr-0.15
	08:XX	12:XX	06:XX	Lr-22.0	04:XX	02:XX	02:XX	Lr-0.15
AB06	08:XX	05:XX	07:XX	Lr-7.0	10:XX	06:XX	01:XX	Lr-0.23
	08:XX	05:XX	07:XX	Lr-7.0	10:XX	06:XX	01:XX	Lr-0.23

2. Farm B (n = 24)

2.1 Gilt (n = 18)

	SLA-1	SLA-2	SLA-3	Haplotype	DRB1	DQB1	DQA	Haplotype
BG01	08:XX	12:XX	06:XX	Lr-22.0	04:XX	02:XX	02:XX	Lr-0.15
	02:XX	11:XX	01:XX	Lr-57.0	11:XX	04:XX	02:XX	Lr-0.26
BG02	06:XX	01:XX	07:XX	Unknown	04:XX	03:XX	02:XX	Lr-0.13
	11:XX	07:XX	03:XX	Lr-25.0	13:XX	09:XX	04:XX+ 05:XX	Lr-0.25
BG03	07:XX	02:XX	04:XX	Lr-32.0	04:XX	07:XX	03:XX	Lr-0.19
	08:XX	12:XX	06:XX	Lr-22.0	04:XX	02:XX	02:XX	Lr-0.15
BG04	08:XX	05:XX	07:XX	Lr-7.0	10:XX	06:XX	01:XX	Lr-0.23

	SLA-1	SLA-2	SLA-3	Haplotype	DRB1	DQB1	DQA	Haplotype
	02:XX	11:XX	01:XX	Lr-57.0	11:XX	04:XX	02:XX	Lr-0.26
BG05	12:XX	10:XX	05:XX	Lr-35.0	10:XX	06:XX	01:XX	Lr-0.23
	02:XX	11:XX	01:XX	Lr-57.0	11:XX	04:XX	02:XX	Lr-0.26
BG06	06:XX	09:XX	05:XX	Lr-29.0	07:XX	02:XX	02:XX	Lr-0.24
	02:XX	11:XX	01:XX	Lr-57.0	11:XX	04:XX	02:XX	Lr-0.26
BG07	08:XX	10:XX	05:XX	Lr-26.0	10:XX	06:XX	01:XX	Lr-0.23
	07:XX	02:XX	04:XX	Lr-32.0	09:XX	08:XX	03:XX	Lr-0.14
BG08	11:XX	07:XX	03:XX	Lr-25.0	04:XX	07:XX	03:XX	Lr-0.19
	11:XX	07:XX	03:XX	Lr-25.0	13:XX	09:XX	04:XX+ 05:XX	Lr-0.25
BG09	07:XX	02:XX	04:XX	Lr-32.0	10:XX	06:XX	01:XX	Lr-0.23
	08:XX	05:XX	07:XX	Lr-7.0	10:XX	06:XX	01:XX	Lr-0.23
BG10	07:XX	02:XX	04:XX	Lr-32.0	04:XX	07:XX	03:XX	Lr-0.19
	07:XX	06:XX	03:XX	Unknown	06:XX	02:XX	04:XX	Unknown
BG11	07:XX	02:XX	04:XX	Lr-32.0	06:XX	07:XX	01:XX	Lr-0.12
	06:XX	09:XX	05:XX	Lr-29.0	06:XX	02:XX	02:XX	Lr-0.22
BG12	08:XX	05:XX	07:XX	Lr-7.0	10:XX	06:XX	01:XX	Lr-0.23
	02:XX	11:XX	01:XX	Lr-57.0	11:XX	04:XX	02:XX	Lr-0.26
BG13	07:XX	02:XX	04:XX	Lr-32.0	04:XX	07:XX	03:XX	Lr-0.19
	14:XX	01:XX	07:XX	Unknown	04:XX	07:XX	03:XX	Lr-0.19
BG14	02:XX	11:XX	01:XX	Lr-57.0	06:XX	07:XX	01:XX	Lr-0.12
	06:XX	06:XX	04:XX	Unknown	11:XX	04:XX	02:XX	Lr-0.26
BG15	08:XX	10:XX	05:XX	Lr-26.0	04:XX	02:XX	02:XX	Lr-0.15
	Blank	02:XX	04:XX	Unknown	04:XX	07:XX	03:XX	Lr-0.19
BG16	07:XX	02:XX	04:XX	Lr-32.0	04:XX	07:XX	03:XX	Lr-0.19
	01:XX	01:XX	01:XX	Lr-1.0	04:XX	02:XX	02:XX	Lr-0.15
BG17	08:XX	05:XX	07:XX	Lr-7.0	04:XX	02:XX	02:XX	Lr-0.15
	08:XX	12:XX	06:XX	Lr-22.0	10:XX	06:XX	01:XX	Lr-0.23
BG18	07:XX	02:XX	04:XX	Lr-32.0	04:XX	07:XX	03:XX	Lr-0.19
	06:XX	01:XX	01:XX	Lr-27.0	06:XX	07:XX	01:XX	Lr-0.12

2.2 Boar (n = 6)

	SLA-1	SLA-2	SLA-3	Haplotype	DRB1	DQB1	DQA	Haplotype
BB01	04:XX	04:XX	04:XX	Lr-4.0	04:XX	07:XX	03:XX	Lr-0.19
	04:XX	04:XX	04:XX	Lr-4.0	04:XX	07:XX	03:XX	Lr-0.19
BB02	04:XX	04:XX	04:XX	Lr-4.0	04:XX	08:XX	03:XX	Lr-0.10
	08:XX	10:XX	05:XX	Lr-26.0	04:XX	08:XX	03:XX	Lr-0.10
BB03	08:XX	04:XX	05:XX	Lr-49.0	02:XX	02:XX	02:XX	Lr-0.2
	08:XX	04:XX	05:XX	Lr-49.0	02:XX	02:XX	02:XX	Lr-0.2
BB04	04:XX	04:XX	04:XX	Lr-4.0	02:XX	02:XX	02:XX	Lr-0.2
	07:XX	02:XX	04:XX	Lr-32.0	02:XX	02:XX	02:XX	Lr-0.2
BB05	04:XX	04:XX	04:XX	Lr-4.0	04:XX	08:XX	03:XX	Lr-0.10
	08:XX	04:XX	05:XX	Lr-49.0	04:XX	08:XX	03:XX	Lr-0.10
BB06	04:XX	04:XX	04:XX	Lr-4.0	02:XX	02:XX	02:XX	Lr-0.2

	SLA-1	SLA-2	SLA-3	Haplotype	DRB1	DQB1	DQA	Haplotype
	08:XX	04:XX	05:XX	Lr-49.0	02:XX	02:XX	02:XX	Lr-0.2

3. Farm C

3.1 Gilt (25)

	SLA-1	SLA-2	SLA-3	Haplotype	DRB1	DQB1	DQA	Haplotype
CG01	12:XX+ 13:XX	10:XX	05:XX	Lr-35.0	01:XX	05:XX	04:XX+ 05:XX	Lr-0.21
	07:XX	05:XX	04:XX	Lr-81.0	10:XX	06:XX	01:XX	Lr-0.23
CG02	08:XX	05:XX	04:XX	Lr-80.0	10:XX	06:XX	01:XX	Lr-0.23
	12:XX+ 13:XX	10:XX	05:XX	Lr-35.0	01:XX	05:XX	04:XX+ 05:XX	Lr-0.21
CG03	01:XX	01:XX	01:XX	Lr-1.0	01:XX	01:XX	01:XX	Lr-0.1
	12:XX+ 13:XX	10:XX	05:XX	Lr-35.0	10:XX	06:XX	01:XX	Lr-0.23
CG04	08:XX	12:XX	06:XX	Lr-22.0	01:XX	05:XX	04:XX+ 05:XX	Lr-0.21
	12:XX+ 13:XX	10:XX	05:XX	Lr-35.0	10:XX	06:XX	01:XX	Lr-0.23
CG05	08:XX	05:XX	04:XX	Lr-80.0	04:XX	08:XX	03:XX	Lr-0.10
	12:XX+ 13:XX	10:XX	05:XX	Lr-35.0	04:XX	08:XX	03:XX	Lr-0.10
CG06	01:XX	01:XX	01:XX	Lr-1.0	01:XX	01:XX	01:XX	Lr-0.1
	12:XX+ 13:XX	10:XX	05:XX	Lr-35.0	10:XX	06:XX	01:XX	Lr-0.23
CG07	08:XX	12:XX	06:XX	Lr-22.0	04:XX	02:XX	02:XX	Lr-0.15
	12:XX+ 13:XX	10:XX	05:XX	Lr-35.0	10:XX	06:XX	01:XX	Lr-0.23
CG08	01:XX	01:XX	01:XX	Lr-1.0	10:XX	06:XX	01:XX	Lr-0.23
	12:XX+ 13:XX	10:XX	05:XX	Lr-35.0	06:XX	07:XX	01:XX	Lr-0.12
CG09	01:XX	01:XX	01:XX	Lr-1.0	01:XX	01:XX	01:XX	Lr-0.1
	09:XX	05:XX	07:XX	Lr-28.0	10:XX	06:XX	01:XX	Lr-0.23
CG10	08:XX	05:XX	04:XX	Lr-80.0	01:XX	05:XX	04:XX+ 05:XX	Lr-0.21
	12:XX+ 13:XX	10:XX	05:XX	Lr-35.0	10:XX	06:XX	01:XX	Lr-0.23
CG11	01:XX	01:XX	01:XX	Lr-1.0	01:XX	01:XX	01:XX	Lr-0.1
	12:XX+ 13:XX	10:XX	05:XX	Lr-35.0	10:XX	06:XX	01:XX	Lr-0.23
CG12	08:XX	12:XX	06:XX	Lr-22.0	07:XX	02:XX	02:XX	Lr-0.24
	12:XX+ 13:XX	10:XX	05:XX	Lr-35.0	10:XX	06:XX	01:XX	Lr-0.23
CG13	01:XX	01:XX	01:XX	Lr-1.0	01:XX	01:XX	01:XX	Lr-0.1
	12:XX+ 13:XX	10:XX	05:XX	Lr-35.0	10:XX	06:XX	01:XX	Lr-0.23
CG14	01:XX	01:XX	01:XX	Lr-1.0	10:XX	06:XX	01:XX	Lr-0.23
	09:XX	05:XX	07:XX	Lr-28.0	10:XX	06:XX	01:XX	Lr-0.23
CG15	08:XX	12:XX	06:XX	Lr-22.0	04:XX	02:XX	02:XX	Lr-0.15
	01:XX	01:XX	01:XX	Lr-1.0	11:XX	04:XX	02:XX	Lr-0.26
CG16	01:XX	01:XX	01:XX	Lr-1.0	10:XX	06:XX	01:XX	Lr-0.23
	08:XX	10:XX	05:XX	Lr-26.0	10:XX	06:XX	01:XX	Lr-0.23
CG17	13:XX	10:XX	05:XX	Lr-35.0	07:XX	02:XX	02:XX	Lr-0.24
	08:XX	10:XX	05:XX	Lr-26.0	10:XX	06:XX	01:XX	Lr-0.23
CG18	07:XX	02:XX	04:XX	Lr-32.0	06:XX	07:XX	01:XX	Lr-0.12
	07:XX	09:XX	05:XX	Lr-37.0	09:XX	09:XX	04:XX+ 05:XX	Lr-0.27

	SLA-1	SLA-2	SLA-3	Haplotype	DRB1	DQB1	DQA	Haplotype
CG19	04:XX	01:XX	04:XX	Unknown	02:XX	04:XX	02:XX	Lr-0.4a
	09:XX	05:XX	07:XX	Lr-28.0	08:XX	02:XX	02:XX	Lr-0.8b
CG20	08:XX	12:XX	06:XX	Lr-22.0	04:XX	02:XX	02:XX	Lr-0.15
	09:XX	05:XX	07:XX	Lr-28.0	10:XX	06:XX	01:XX	Lr-0.23
CG21	07:XX	05:XX	06:XX	Lr-21.0	01:XX	01:XX	01:XX	Lr-0.1
	09:XX	05:XX	07:XX	Lr-28.0	08:XX	02:XX	02:XX	Lr-0.8b
CG22	08:XX	12:XX	06:XX	Lr-22.0	01:XX	01:XX	01:XX	Lr-0.1
	06:XX	02:XX	04:XX	Unknown	08:XX	02:XX	02:XX	Lr-0.8b
CG23	08:XX	07:XX	03:XX	Lr-82.0	02:XX	02:XX	02:XX	Lr-0.2
	08:XX	09:XX	03:XX	Lr-58.0	06:XX	07:XX	01:XX	Lr-0.12
CG24	08:XX	05:XX	06:XX	Lr-6.0	02:XX	02:XX	02:XX	Lr-0.2
	04:XX	14:XX	04:XX	Unknown	06:XX	07:XX	01:XX	Lr-0.12
CG25	11:XX	13:XX	05:XX	Potential	01:XX	01:XX	01:XX	Lr-0.1
	07:XX	05:XX	06:XX	Lr-21.0	09:XX	09:XX	04:XX+ 05:XX	Lr-0.27

3.2 **Boar (n = 10)**

	SLA-1	SLA-2	SLA-3	Haplotype	DRB1	DQB1	DQA	Haplotype
CB01	08:XX	10:XX	05:XX	Lr-26.0	02:XX	02:XX	02:XX	Lr-0.2
	08:XX	10:XX	05:XX	Lr-26.0	02:XX	02:XX	02:XX	Lr-0.2
CB02	04:XX	06:XX	03:XX	Lr-18.0	06:XX	07:XX	01:XX	Lr-0.12
	Blank	14:XX	04:XX	Unknown	05:XX	02:XX	02:XX	Lr-0.5
CB03	08:XX	05:XX	07:XX	Lr-7.0	10:XX	06:XX	01:XX	Lr-0.23
	08:XX	05:XX	07:XX	Lr-7.0	10:XX	06:XX	01:XX	Lr-0.23
CB04	04:XX	04:XX	04:XX	Lr-4.0	02:XX	02:XX	02:XX	Lr-0.2
	04:XX	04:XX	04:XX	Lr-4.0	02:XX	02:XX	02:XX	Lr-0.2
CB05	04:XX	09:XX	04:XX	Potential	01:XX	10:XX	04:XX	Lr-0.36
	08:XX	10:XX	05:XX	Lr-26.0	09:XX	09:XX	04:XX+ 05:XX	Lr-0.27
CB06	04:XX	04:XX	04:XX	Lr-4.0	02:XX	02:XX	02:XX	Lr-0.2
	04:XX	04:XX	04:XX	Lr-4.0	09:XX	09:XX	04:XX+ 05:XX	Lr-0.27
CB07	08:XX	10:XX	05:XX	Lr-26.0	10:XX	06:XX	01:XX	Lr-0.23
	07:XX	05:XX	06:XX	Lr-21.0	06:XX	02:XX	02:XX	Lr-0.22
CB08	04:XX	04:XX	04:XX	Lr-4.0	02:XX	02:XX	02:XX	Lr-0.2
	04:XX	09:XX	04:XX	Potential	02:XX	02:XX	02:XX	Lr-0.2
CB09	04:XX	09:XX	04:XX	Potential	02:XX	02:XX	02:XX	Lr-0.2
	08:XX	10:XX	05:XX	Lr-26.0	09:XX	09:XX	04:XX+ 05:XX	Lr-0.27
CB10	04:XX	09:XX	04:XX	Potential	02:XX	02:XX	02:XX	Lr-0.2
	08:XX	10:XX	05:XX	Lr-26.0	09:XX	09:XX	04:XX+ 05:XX	Lr-0.27

4. **Farm D (32)**

4.1 **Gilt (22)**

	SLA-1	SLA-2	SLA-3	Haplotype	DRB1	DQB1	DQA	Haplotype
DG01	07:XX	02:XX	04:XX	Lr-32.0	09:XX	09:XX	04:XX+ 05:XX	Lr-0.27
	02:XX	02:XX	04:XX	Lr-2.0	10:XX	02:XX	03:XX	Potential

	SLA-1	SLA-2	SLA-3	Haplotype	DRB1	DQB1	DQA	Haplotype
DG02	02:XX	11:XX	01:XX	Lr-57.0	09:XX	09:XX	04:XX+ 05:XX	Lr-0.27
	08:XX	05:XX	07:XX	Lr-7.0	10:XX	02:XX	03:XX	Potential
DG03	01:XX	01:XX	01:XX	Lr-1.0	01:XX	01:XX	01:XX	Lr-0.1
	08:XX	05:XX	07:XX	Lr-7.0	10:XX	06:XX	01:XX	Lr-0.23
DG04	08:XX	05:XX	07:XX	Lr-7.0	10:XX	06:XX	01:XX	Lr-0.23
	Blank	09:XX	05:XX	Lr-29.0	07:XX	02:XX	02:XX	Lr-0.24
DG05	07:XX	02:XX	04:XX	Lr-32.0	04:XX	07:XX	03:XX	Lr-0.19
	02:XX	02:XX	04:XX	Lr-2.0	10:XX	06:XX	01:XX	Lr-0.23
DG06	02:XX	11:XX	01:XX	Lr-57.0	07:XX	02:XX	02:XX	Lr-0.24
	08:XX	05:XX	07:XX	Lr-7.0	10:XX	06:XX	01:XX	Lr-0.23
DG07	08:XX	12:XX	06:XX	Lr-22.0	04:XX	02:XX	02:XX	Lr-0.15
	08:XX	10:XX	05:XX	Lr-26.0	07:XX	02:XX	02:XX	Lr-0.24
DG08	02:XX	11:XX	01:XX	Lr-57.0	07:XX	02:XX	02:XX	Lr-0.24
	08:XX	05:XX	07:XX	Lr-7.0	10:XX	06:XX	01:XX	Lr-0.23
DG09	Blank	09:XX	05:XX	Lr-29.0	10:XX	06:XX	01:XX	Lr-0.23
	08:XX	05:XX	07:XX	Lr-7.0	07:XX	02:XX	02:XX	Lr-0.24
DG10	06:XX	09:XX	05:XX	Lr-29.0	10:XX	06:XX	01:XX	Lr-0.23
	08:XX	10:XX	05:XX	Lr-26.0	07:XX	02:XX	02:XX	Lr-0.24
DG11	08:XX	10:XX	05:XX	Lr-26.0	10:XX	06:XX	01:XX	Lr-0.23
	08:XX	12:XX	06:XX	Lr-22.0	04:XX	02:XX	02:XX	Lr-0.15
DG12	08:XX	05:XX	07:XX	Lr-7.0	10:XX	06:XX	01:XX	Lr-0.23
	08:XX	12:XX	06:XX	Lr-22.0	04:XX	02:XX	02:XX	Lr-0.15
DG13	08:XX	12:XX	06:XX	Lr-22.0	04:XX	02:XX	02:XX	Lr-0.15
	08:XX	12:XX	06:XX	Lr-22.0	04:XX	02:XX	02:XX	Lr-0.15
DG14	01:XX	01:XX	01:XX	Lr-1.0	01:XX	01:XX	01:XX	Lr-0.1
	08:XX	10:XX	05:XX	Lr-26.0	10:XX	06:XX	01:XX	Lr-0.23
DG15	07:XX	02:XX	04:XX	Lr-32.0	01:XX	01:XX	01:XX	Lr-0.1
	02:XX	02:XX	04:XX	Lr-2.0	06:XX	07:XX	01:XX	Lr-0.12
DG16	07:XX	02:XX	04:XX	Lr-32.0	04:XX	07:XX	03:XX	Lr-0.19
	01:XX	01:XX	01:XX	Lr-1.0	07:XX	02:XX	02:XX	Lr-0.24
DG17	07:XX	02:XX	04:XX	Lr-32.0	04:XX	07:XX	03:XX	Lr-0.19
	02:XX	11:XX	01:XX	Lr-57.0	11:XX	04:XX	02:XX	Lr-0.26
DG18	07:XX	02:XX	04:XX	Lr-32.0	11:XX	04:XX	02:XX	Lr-0.26
	02:XX	11:XX	01:XX	Lr-57.0	02:XX	02:XX	02:XX	Lr-0.2
DG19	02:XX	11:XX	01:XX	Lr-57.0	04:XX	02:XX	02:XX	Lr-0.15
	02:XX	11:XX	01:XX	Lr-57.0	11:XX	04:XX	02:XX	Lr-0.26
DG20	01:XX	01:XX	01:XX	Lr-1.0	01:XX	01:XX	01:XX	Lr-0.1
	06:XX	01:XX	01:XX	Lr-27.0	07:XX	02:XX	02:XX	Lr-0.24
DG21	07:XX	02:XX	04:XX	Lr-32.0	06:XX	07:XX	01:XX	Lr-0.12
	07:XX	02:XX	04:XX	Lr-32.0	06:XX	07:XX	01:XX	Lr-0.12
DG22	01:XX	01:XX	01:XX	Lr-1.0	04:XX	02:XX	02:XX	Lr-0.15
	08:XX	12:XX	06:XX	Lr-22.0	01:XX	01:XX	01:XX	Lr-0.1

4.2 Boar (10)

	SLA-1	SLA-2	SLA-3	Haplotype	DRB1	DQB1	DQA	Haplotype
DB01	04:XX	04:XX	04:XX	Lr-4.0	02:XX	02:XX	02:XX	Lr-0.2
	04:XX	04:XX	04:XX	Lr-4.0	02:XX	02:XX	02:XX	Lr-0.2
DB02	04:XX	04:XX	04:XX	Lr-4.0	02:XX	02:XX	02:XX	Lr-0.2
	04:XX	04:XX	04:XX	Lr-4.0	02:XX	02:XX	02:XX	Lr-0.2
DB03	04:XX	04:XX	04:XX	Lr-4.0	02:XX	02:XX	02:XX	Lr-0.2
	04:XX	04:XX	04:XX	Lr-4.0	02:XX	02:XX	02:XX	Lr-0.2
DB04	04:XX	04:XX	04:XX	Lr-4.0	02:XX	02:XX	02:XX	Lr-0.2
	08:XX	10:XX	05:XX	Lr-26.0	02:XX	02:XX	02:XX	Lr-0.2
DB05	04:XX	04:XX	04:XX	Lr-4.0	02:XX	02:XX	02:XX	Lr-0.2
	04:XX	04:XX	04:XX	Lr-4.0	02:XX	02:XX	02:XX	Lr-0.2
DB06	04:XX	04:XX	04:XX	Lr-4.0	02:XX	02:XX	02:XX	Lr-0.2
	08:XX	10:XX	05:XX	Lr-26.0	13:XX	09:XX	04:XX+ 05:XX	Lr-0.25
DB07	07:XX	02:XX	04:XX	Lr-32.0	02:XX	02:XX	02:XX	Lr-0.2
	04:XX	04:XX	04:XX	Lr-4.0	02:XX	02:XX	02:XX	Lr-0.2
DB08	07:XX	02:XX	04:XX	Lr-32.0	02:XX	02:XX	02:XX	Lr-0.2
	04:XX	04:XX	04:XX	Lr-4.0	02:XX	02:XX	02:XX	Lr-0.2
DB09	04:XX	04:XX	04:XX	Lr-4.0	02:XX	02:XX	02:XX	Lr-0.2
	08:XX	10:XX	05:XX	Lr-26.0	02:XX	02:XX	02:XX	Lr-0.2
DB10	04:XX	04:XX	04:XX	Lr-4.0	02:XX	02:XX	02:XX	Lr-0.2
	04:XX	04:XX	04:XX	Lr-4.0	02:XX	02:XX	02:XX	Lr-0.2

5. Farm E gilt (n = 35)

	SLA-1	SLA-2	SLA-3	Haplotype	DRB1	DQB1	DQA	Haplotype
EG01	11:XX	13:XX	05:XX	Potential	04:XX	02:XX	02:XX	Lr-0.15
	08:XX	12:XX	06:XX	Lr-22.0	09:XX	09:XX	04:XX+ 05:XX	Lr-0.27
EG02	11:XX	04:XX	04:XX	Lr-43.0	04:XX	06:XX	01:XX	Unknown
	07:XX	09:XX	05:XX	Lr-37.0	09:XX	09:XX	04:XX+ 05:XX	Lr-0.27
EG03	07:XX	09:XX	05:XX	Lr-37.0	04:XX	08:XX	03:XX	Lr-0.10
	12:XX	10:XX	05:XX	Lr-35.0	09:XX	08:XX	03:XX	Lr-0.14
EG04	07:XX	09:XX	05:XX	Lr-37.0	10:XX	06:XX	01:XX	Lr-0.23
	08:XX	12:XX	06:XX	Lr-22.0	09:XX	09:XX	04:XX+ 05:XX	Lr-0.27
EG05	08:XX	09:XX	05:XX	Lr-29.0	05:XX	08:XX	01:XX	Lr-0.6
	11:XX	04:XX	04:XX	Lr-43.0	09:XX	09:XX	04:XX+ 05:XX	Lr-0.27
EG06	08:XX	10:XX	05:XX	Lr-26.0	10:XX	06:XX	01:XX	Lr-0.23
	11:XX	07:XX	03:XX	Lr-25.0	13:XX	09:XX	04:XX+ 05:XX	Lr-0.25
EG07	08:XX	10:XX	05:XX	Lr-26.0	09:XX	09:XX	03:XX	Unknown
	07:XX	02:XX	04:XX	Lr-32.0	06:XX	07:XX	01:XX	Lr-0.12
EG08	08:XX	10:XX	05:XX	Lr-26.0	05:XX	08:XX	01:XX	Lr-0.6
	11:XX	09:XX	03:XX	Lr-70.0	09:XX	09:XX	04:XX+ 05:XX	Lr-0.27
EG09	11:XX	07:XX	03:XX	Lr-25.0	13:XX	09:XX	04:XX	Lr-0.25
	07:XX	09:XX	05:XX	Lr-37.0	09:XX	09:XX	04:XX+ 05:XX	Lr-0.27

	SLA-1	SLA-2	SLA-3	Haplotype	DRB1	DQB1	DQA	Haplotype
EG10	12:XX+ 13:XX	10:XX	05:XX	Lr-35.0	06:XX	07:XX	01:XX	Lr-0.12
	12:XX+ 13:XX	10:XX	05:XX	Lr-35.0	10:XX	02:XX	03:XX	Potential
EG11	02:XX	11:XX	01:XX	Lr-57.0	11:XX	04:XX	02:XX	Lr-0.26
	07:XX	09:XX	05:XX	Lr-37.0	07:XX	02:XX	02:XX	Lr-0.24
EG12	07:XX	02:XX	04:XX	Lr-32.0	06:XX	07:XX	01:XX	Lr-0.12
	11:XX	Blank	05:XX	Lr-63.0	09:XX	09:XX	04:XX+ 05:XX	Lr-0.27
EG13	07:XX	09:XX	05:XX	Lr-37.0	05:XX	08:XX	01:XX	Lr-0.6
	Blank	11:XX	04:XX	Unknown	07:XX	02:XX	02:XX	Lr-0.24
EG14	08:XX	10:XX	05:XX	Lr-26.0	10:XX	06:XX	01:XX	Lr-0.23
	11:XX	14:XX	Blank	Unknown	04:XX	07:XX	03:XX	Lr-0.19
EG15	Blank	01:XX	01:XX	Unknown	04:XX	07:XX	03:XX	Lr-0.19
	Blank	09:XX	05:XX	Lr-29.0	07:XX	02:XX	02:XX	Lr-0.24
EG16	02:XX	11:XX	01:XX	Lr-57.0	11:XX	04:XX	02:XX	Lr-0.26
	06:XX	09:XX	05:XX	Lr-29.0	07:XX	02:XX	02:XX	Lr-0.24
EG17	08:XX	12:XX	06:XX	Lr-22.0	04:XX	08:XX	03:XX	Lr-0.10
	08:XX	10:XX	05:XX	Lr-26.0	04:XX	02:XX	02:XX	Lr-0.15
EG18	06:XX	09:XX	05:XX	Lr-29.0	07:XX	02:XX	02:XX	Lr-0.24
	11:XX	07:XX	03:XX	Lr-25.0	13:XX	09:XX	04:XX+ 05:XX	Lr-0.25
EG19	07:XX	02:XX	04:XX	Lr-32.0	07:XX	02:XX	02:XX	Lr-0.24
	06:XX	09:XX	05:XX	Lr-29.0	06:XX	07:XX	01:XX	Lr-0.12
EG20	08:XX	12:XX	06:XX	Lr-22.0	04:XX	02:XX	02:XX	Lr-0.15
	11:XX	01:XX	01:XX	Potential	04:XX	07:XX	03:XX	Lr-0.19
EG21	07:XX	02:XX	04:XX	Lr-32.0	07:XX	02:XX	02:XX	Lr-0.24
	06:XX	09:XX	05:XX	Lr-29.0	06:XX	07:XX	01:XX	Lr-0.12
EG22	07:XX	02:XX	04:XX	Lr-32.0	04:XX	07:XX	03:XX	Lr-0.19
	02:XX	02:XX	04:XX	Lr-2.0	10:XX	06:XX	01:XX	Lr-0.23
EG23	06:XX	09:XX	05:XX	Lr-29.0	07:XX	02:XX	02:XX	Lr-0.24
	08:XX	10:XX	05:XX	Lr-26.0	06:XX	07:XX	01:XX	Lr-0.12
EG24	11:XX	01:XX	01:XX	Potential	04:XX	02:XX	02:XX	Lr-0.15
	08:XX	12:XX	06:XX	Lr-22.0	04:XX	07:XX	03:XX	Lr-0.19
EG25	08:XX	10:XX	05:XX	Lr-26.0	02:XX	02:XX	02:XX	Lr-0.2
	11:XX	09:XX	03:XX	Lr-70.0	09:XX	09:XX	04:XX+ 05:XX	Lr-0.27
EG26	08:XX	12:XX	06:XX	Lr-22.0	04:XX	02:XX	02:XX	Lr-0.15
	08:XX	12:XX	06:XX	Lr-22.0	04:XX	02:XX	02:XX	Lr-0.15
EG27	07:XX	02:XX	04:XX	Lr-32.0	06:XX	07:XX	01:XX	Lr-0.12
	07:XX	09:XX	05:XX	Lr-37.0	09:XX	09:XX	04:XX+ 05:XX	Lr-0.27
EG28	07:XX	11:XX	04:XX	Lr-76.0	04:XX	02:XX	02:XX	Lr-0.15
	08:XX	12:XX	06:XX	Lr-22.0	05:XX	08:XX	01:XX	Lr-0.6
EG29	07:XX	02:XX	04:XX	Lr-32.0	06:XX	07:XX	01:XX	Lr-0.12
	06:XX	09:XX	05:XX	Lr-29.0	09:XX	09:XX	04:XX+ 05:XX	Lr-0.27
EG30	11:XX	07:XX	03:XX	Lr-25.0	13:XX	09:XX	04:XX	Lr-0.25

	SLA-1	SLA-2	SLA-3	Haplotype	DRB1	DQB1	DQA	Haplotype
	Blank	13:XX	05:XX	Unknown	09:XX	09:XX	04:XX+ 05:XX	Lr-0.27
EG31	11:XX	04:XX	04:XX	Lr-43.0	09:XX	09:XX	04:XX+ 05:XX	Lr-0.27
	08:XX	04:XX	05:XX	Lr-49.0	09:XX	09:XX	04:XX+ 05:XX	Lr-0.27
EG32	07:XX	09:XX	05:XX	Lr-37.0	09:XX	09:XX	04:XX+ 05:XX	Lr-0.27
	11:XX	04:XX	04:XX	Lr-43.0	09:XX	09:XX	04:XX+ 05:XX	Lr-0.27
EG33	07:XX	02:XX	04:XX	Lr-32.0	06:XX	07:XX	01:XX	Lr-0.12
	11:XX	04:XX	04:XX	Lr-43.0	09:XX	09:XX	04:XX+ 05:XX	Lr-0.27
EG34	Blank	01:XX	04:XX	Unknown	06:XX	07:XX	01:XX	Lr-0.12
	12:XX+ 13:XX	10:XX	05:XX	Lr-35.0	10:XX	02:XX	03:XX	Potential
EG35	06:XX	09:XX	05:XX	Lr-29.0	13:XX	09:XX	04:XX	Lr-0.25
	11:XX	07:XX	03:XX	Lr-25.0	07:XX	02:XX	02:XX	Lr-0.24

Supplementary Table 2 Frequency and SLA class I and II haplotypes of homozygous animals in the study population

	SLA class I		SLA class II		Complete haplotype*	
**Farm**	**Boars**	**Gilts**	**Boars**	**Gilts**	**Boars**	**Gilts**
**A (32)**	4/6	1/26	4/6	0/26	4/6	0/26
	Lr-4.0<br>Lr-7.0<br>Lr-22.0<br>Lr-32.0	Lr-25.0	Lr-0.2<br>Lr-0.12<br>Lr-0.15<br>Lr-0.23	-	Lr-4.2<br>Lr-7.23<br>Lr-22.15<br>Lr-32.12	-
**B (24)**	2/6	1/18	6/6	1/18	2/6	0/18
	Lr-4.0<br>Lr-49.0	Lr-25.0	Lr-0.2 (3)<br>Lr-0.10 (2)<br>Lr-0.19	Lr-0.19	Lr-4.19<br>Lr-49.2	-
**C (35)**	4/10	0/25	4/10	3/25	3/10	0/25
	Lr-4.0 (2)<br>Lr-7.0<br>Lr-26.0	-	Lr-0.2 (3)<br>Lr-0.23	Lr-0.10<br>Lr-0.23 (2)	Lr-4.2<br>Lr-7.23<br>Lr-26.2	-
**D (32)**	5/10	3/22	9/10	2/22	5/10	2/22
	Lr-4.0 (5)	Lr-22.0<br>Lr-32.0<br>Lr-57.0	Lr-0.2 (9)	Lr-0.12<br>Lr-0.15	Lr-4.2 (5)	Lr-22.15<br>Lr-32.12
**E (35)**	n/a**	2/35	n/a	3/35	n/a	1/35
		Lr-22.0<br>Lr-35.0		Lr-0.15<br>Lr-0.27 (2)		Lr-22.15
**total**	15/32	7/126	23/32	9/126	14/32	3/126

*Homozygous in all 6 SLA loci **Boars from farm E were not enrolled in this study.

Supplementary Table 3 Frequency of SLA class I allelic groups of Thai pigs in comparison with other populations

	Allelic group*	Thai boars[a] (n=32)	Thai gilts[b] (n=126)	Canadian Landrace (n=22)	Canadian Yorkshire (n=15)	German Landrace (n=155)	Danish crossbreeds[c] (n=100)	US PCV+KSU[d] (n=101)	US Big Pig[e] (n=101)
**SLA-1**	01:XX		7.5%		10.0%	0.8%	3.0%	17.8%	26.2%
	02:XX		8.7%			0.8%	14.4%	1.5%	
	04:XX	53.1%	1.2%	4.6%	20.0%	0.8%	19.3%	30.7%	22.8%
	05:XX								
	06:XX		6.0%					5.4%	0.5%
	07:XX	10.9%	23.8%	15.9%	6.7%	6.9%	13.9%		2.5%
	08:XX	32.8%	29.4%	18.2%	43.3%	23.9%	32.7%	11.8%	24.3%
	09:XX		2.0%	9.1%		0.8%		2.4%	5.4%
	10:XX								
	11:XX		9.5%		13.3%	15.3%	1.5%	18.3%	0.5%
	12:XX		1.2%	2.3%		5.4%			
	13:XX		6.4%		6.7%				
	14:XX		0.4%	29.6%		16.2%	1.0%		
	16:XX						1.0%		
	17:XX	1.6%	0.8%						
	Blank**	1.6%	3.2%	20.5%		29.2%		6.4%	16.4%
**SLA-2**	01:XX		11.1%		10.0%	0.8%	3.0%	28.7%	35.1%
	02:XX	9.4%	20.6%	25.0%		3.1%	25.7%	1.5%	0.5%
	03:XX								
	04:XX	51.6%	2.8%	4.6%	20.0%	6.2%	19.3%	33.2%	21.3%
	05:XX	7.8%	9.9%	36.4%	3.3%	29.2%	10.9%	8.9%	7.0%
	06:XX	1.6%	1.6%	15.9%		22.3%	9.9%	3.5%	0.5%
	07:XX		4.8%			6.9%	1.5%	12.9%	0.5%
	08:XX					5.4%	8.4%		1.5%
	09:XX	6.3%	11.1%		6.7%		1.0%		3.0%
	10:XX	17.2%	13.9%	18.2%	46.7%	10.8%	17.3%	2.9%	11.4%
	11:XX		6.8%				2.5%		19.3%
	12:XX	4.7%	15.1%				7.4%	5.45%	
	13:XX		1.2%						
	14:XX	1.6%	0.8%						
	15:XX					1.5%			
	17:XX					1.5%			
	Blank**		0.4%			4.6%		2.9%	
**SLA-3**	01:XX		15.5%		10.0%	0.8%	5.4%	23.3%	28.2%
	03:XX	1.6%	6.8%		3.3%	13.1%	8.9%	16.3%	0.5%
	04:XX	62.5%	27.4%	45.5%	20.0%	40.8%	48.0%	34.7%	22.3%
	05:XX	21.9%	25.0%	18.2%	63.3%	16.9%	11.4%	5.8%	16.4%
	06:XX	6.3%	16.3%	2.3%	3.3%	12.3%	8.4%	7.9%	25.8%
	07:XX	7.8%	8.7%	34.1%		16.2%	17.8%	11.9%	6.9%
	08:XX								
	Blank**		0.4%						

*The study primer sets cannot identify SLA-1*03:XX, SLA-1*15:XX, SLA-2*16:XX and SLA-3*02:XX. **Blank indicates alleles that cannot be identified with the study primer sets. [a]Thai boars were 100%Duroc. [b]Thai gilts were either (50%Landrace/50%Yorkshire, n = 113) or (25%L/75%Y, n = 13). [c]Danish crossbreeds were 50%LY/50%D (Pedersen et al., 2014). [d]US PCV+KSU were 50%LY/50%D crossbreeds. [e]US Big Pig were 50%L/50%Y crossbreeds (Ho et al., 2009).

Supplementary Table 4 Frequency of SLA class I haplotypes of Thai pigs in comparison with other populations

Haplotype	Thai boars[a] (n=32)	Thai gilts[b] (n=126)	Canadian Landrace (n=22)	Canadian Yorkshire (n=15)	German Landrace (n=155)	Danish crossbred[c] (n=100)	US PCV+KSU[d] (n=101)	US Big Pig[e] (n=101)
Lr-1.0		7.5%		10.0%	0.8%	3.0%	17.8%	25.7%
Lr-2.0		2.8%			0.8%	11.9%	1.5%	
Lr-4.0	45.3%	0.4%	4.5%	20.0%	0.8%	19.3%	30.7%	21.3%
Lr-5.0								1.5%
Lr-6.0		0.4%	2.3%		10.8%	1.0%		5.0%
Lr-7.0	6.3%	5.2%	13.6%		2.3%	9.4%	3.5%	
Lr-11.0							2.9%	
Lr-17.0		0.4%						
Lr-18.0	1.6%							
Lr-21.0	1.6%	0.8%		3.3%			2.5%	1.5%
Lr-22.0	4.7%	14.7%				7.4%	5.5%	19.3%
Lr-24.0					1.5%	1.5%		
Lr-25.0		4.4%			6.9%	1.5%	12.9%	0.5%
Lr-26.0	15.6%	6.0%	2.3%	40.0%	0.8%	5.0%	2.9%	
Lr-27.0		0.8%					5.5%	0.5%
Lr-28.0		2.0%	20.5%		0.8%		2.5%	5.4%
Lr-29.0		5.6%				1.0%		3.0%
Lr-30.0								2.0%
Lr-31.0							2.9%	1.5%
Lr-32.0	9.4%	17.1%	15.9%		2.3%	13.9%		0.5%
Lr-33.0								0.5%
Lr-34.0					13.8%	0.5%		0.5%
Lr-35.0		7.1%	2.3%	6.7%				
Lr-37.0		4.4%		3.3%	5.4%			
Lr-39.0					4.6%	3.0%		11.4%
Lr-40.0						1.0%		
Lr-43.0		2.0%			5.4%		2.5%	
Lr-45.0					5.4%	8.4%		
Lr-47.0					1.5%			
Lr-49.0	6.3%	0.4%			4.6%	1.5%		
Lr-50.0		0.4%						
Lr-52.0					7.7%			
Lr-56.0					1.5%			
Lr-57.0		6.0%				3.0%		
Lr-58.0		0.4%		3.3%				
Lr-59.0					1.5%		2.9%	
Lr-61.0					4.6%	6.9%	3.5%	
Lr-62.0			15.9%		16.2%	1.0%		
Lr-63.0		0.4%		13.3%				
Lr-64.0			13.6%					
Lr-70.0		0.8%						
Lr-73.0			9.1%					
Lr-76.0		0.4%						
Lr-80.0		1.2%						
Lr-81.0		0.4%						
Lr-82.0		0.4%						

*The study primer sets cannot identify SLA-1*03:XX, SLA-1*15:XX, SLA-2*16:XX and SLA-3*02:XX. [a]Thai boars were 100%Duroc. [b]Thai gilts were either (50%Landrace/50%Yorkshire, n = 113) or (25%L/75%Y, n = 13). [c]Danish crossbreeds were 50%LY/50%D (Pedersen et al., 2014). [d]US PCV+KSU were 50%LY/50%D crossbreeds. [e]US Big Pig were 50%L/50%Y crossbreeds (Ho et al., 2009).

Supplementary Table 5 Frequency of SLA class II allelic groups of Thai pigs in comparison with other populations

	Allelic group	Thai boar[a] (n=32)	Thai gilt[b] (n=126)	Canadian Landrace (n=22)	Canadian Yorkshire (n=15)	German Landrace (n=155)	US PCV+KSU[c] (n=101)	US Big Pig[d] (n=93)
**DRB1**	01:XX	1.6%	8.7%			12.3%	21.8%	17.2%
	02:XX	57.8%	2.4%		23.3%		23.3%	26.3%
	03:XX							
	04:XX	15.6%	24.6%		10.0%	7.7%	27.2%	22.6%
	05:XX		1.6%	4.6%			5.0%	
	06:XX	3.1%	17.1%	43.2%	3.3%	40.8%	11.4%	2.7%
	07:XX		6.7%		3.3%			3.8%
	08:XX	4.7%	1.2%	11.4%		3.1%	2.5%	
	09:XX	7.8%	9.9%		13.3%	7.7%	5.5%	
	10:XX	7.8%	17.9%	40.9%	46.7%	10.8%		14.0%
	11:XX		4.8%			6.2%	3.5%	11.8%
	12:XX							
	13:XX	1.6%	3.6%			6.9%	1.5%	
	14:XX							
	Blank*					4.6%		
**DQB1**	01:XX		6.8%				16.3%	17.2%
	02:XX	65.6%	25.0%	15.9%	20.0%	3.1%	44.6%	30.1%
	03:XX		3.2%				6.4%	3.2%
	04:XX		6.0%		20.0%	5.4%	3.5%	31.2%
	05:XX		1.6%			13.1%	5.5%	
	06:XX	7.8%	16.7%	40.9%	46.7%	10.8%		14.0%
	07:XX	9.9%	17.5%	45.5%		48.5%	15.8%	2.7%
	08:XX	7.8%	4.0%			7.7%	4.6%	
	09:XX	7.8%	18.7%		13.3%	11.5%	3.0%	1.6%
	10:XX	1.6%						
	Blank*		0.8%		13.3%			
**DQA**	01:XX	12.5%	40.9%			51.5%	23.3%	33.3%
	02:XX	65.6%	31.8%			9.2%	51.5%	64.5%
	03:XX	12.5%	13.5%			15.4%	14.9%	0.5%
	04:XX+ 05:XX	9.4%	13.9%			23.8%	8.4%	1.6%

*Blank indicates alleles that cannot be identified with the study primer sets. [a]Thai boars were 100%Duroc. [b]Thai gilts were either (50%Landrace/50%Yorkshire, n = 113) or (25%L/75%Y, n = 13). [c]US PCV+KSU were 50%LY/50%D crossbreeds. [d]US Big Pig were 50%L/50%Y crossbreeds (Ho et al., 2010).

Supplementary Table 6 Frequency of SLA class II haplotypes of Thai pigs in comparison with other populations

Haplotype	Thai boar[a] (n=32)	Thai gilt[b] (n=126)	Canadian Landrace (n=22)	Canadian Yorkshire (n=15)	German Landrace (n=155)	US PCV+KSU[c] (n=101)	US Big Pig[d] (n=93)
Lr-0.1		6.8%				16.3%	17.2%
Lr-0.2	57.8%	2.0%		3.3%		23.3%	6.9%
Lr-0.4		0.4%		20%			19.4%
Lr-0.5	1.6%		4.5%			4.9%	
Lr-0.6		1.6%					
Lr-0.8		1.2%	11.4%		3.1%	2.5%	
Lr-0.10	6.3%	1.6%				1.9%	
Lr-0.11		0.8%					
Lr-0.12	4.7%	15.9%	38.6%		40.8%	5.9%	2.2%
Lr-0.13		0.4%				3.5%	3.2%
Lr-0.14	1.6%	0.8%			7.7%	2.5%	
Lr-0.15	4.7%	13.9%		10.0%		11.4%	18.8%
Lr-0.19		8.3%			7.7%	10.4%	0.5%
Lr-0.20						2.9%	
Lr-0.21		1.6%			12.3%	5.5%	
Lr-0.22	1.6%	0.8%		3.3%		2.5%	
Lr-0.23	7.8%	16.3%	45.5%	46.7%	10.8%		13.9%
Lr-0.24		8.3%		3.3%			3.8%
Lr-0.25	1.6%	3.6%			6.9%		1.6%
Lr-0.26		4.8%			5.4%	3.5%	11.8%
Lr-0.27	6.3%	8.0%		13.3%		2.9%	
Lr-0.29					4.6%		
Lr-0.30					0.8%		
Lr-0.36	1.6%						

[a]Thai boars were 100%Duroc. [b]Thai gilts were either (50%Landrace/50%Yorkshire, n = 113) or (25%L/75%Y, n = 13). [c]US PCV+KSU were 50%LY/50%D crossbreeds. [d]US Big Pig were 50%L/50%Y crossbreeds (Ho et al., 2010).

Supplementary Table 7 Common SLA haplotypes of Thai pigs in comparison with other populations

Haplotype	Thai boar[a] (n=32)	Thai gilt[b] (n=126)	Canadian Landrace (n=22)	Canadian Yorkshire (n=15)	German Landrace (n=155)
Lr-1.1		5.2%			
Lr-4.2	37.5%	0.4%			
Lr-6.12					10.8%
Lr-7.23	6.25%	4.8%			
Lr-22.15	4.69%	12.7%			
Lr-25.25		3.6%			6.9%
Lr-26.23	1.56%	3.2%	4.5%	40%	
Lr-28.23		1.2%	13.6%		0.8%
Lr-32.12	3.13%	12.7%	15.9%		2.3%
Lr-34.21					12.3%
Lr-35.23		4.8%	2.3%	6.7%	3.8%
Lr-62.12					16.2%
Lr-64.23			13.6%		

[a]Thai boars were 100%Duroc. [b]Thai gilts were either (50%Landrace/50%Yorkshire, n = 113) or (25%L/75%Y, n = 13).

Supplementary Table 8 Shared SLA class I and II haplotypes of Thai pigs with other populations

SLA class I haplotype	Population	SLA class II haplotype	Population
Lr-1.0	Austrian Pietrain<br>Canadian Yorkshire<br>German Landrace<br>Austrian crossbreeds<br>Danish crossbreeds<br>US KSU, PCV, Big Pig	Lr-0.1	Austrian Pietrain<br>Meishan<br>US KSU, PCV, Big Pig<br>Korean native pigs
Lr-2.0	Danish crossbreeds	Lr-0.2	Canadian Yorkshire<br>US KSU, PCV, Big Pig
Lr-4.0	Canadian Landrace&Yorkshire<br>Meishan<br>Austrian crossbreeds<br>Danish crossbreeds<br>US KSU, PCV, Big Pig	Lr-0.4	Canadian Yorkshire<br>US Big Pig
Lr-6.0	Austrian crossbreeds<br>Danish crossbreeds<br>US Big Pig	Lr-0.5	Canadian Landrace&Yorkshire<br>US PCV
Lr-7.0	Canadian Landrace<br>Austrian crossbreeds<br>Danish crossbreeds<br>US PCV<br>Korean native pigs	Lr-0.6	Austrian Pietrain
Lr-17.0	Clawn minipig<br>Microminipig	Lr-0.8	Canadian Landrace<br>Austrian crossbreeds<br>US PCV
Lr-18.0	Meishan	Lr-0.10	US PCV
Lr-21.0	Canadian Yorkshire	Lr-0.11	Microminipig
Lr-22.0	Danish crossbreeds<br>US KSU, PCV, Big Pig	Lr-0.12	Austrian Pietrain<br>Canadian Landrace<br>US PCV, Big Pig
Lr-25.0	Austrian Pietrain<br>Austrian crossbreeds<br>Danish crossbreeds<br>US KSU, PCV, Big pig	Lr-0.13	Austrian Pietrain<br>Microminipig<br>US PCV, Big Pig, Meishan<br>Korean native pigs
Lr-26.0	Austrian Pietrain<br>Canadian Landrace&Yorkshire<br>Austrian crossbreeds<br>Danish crossbreeds<br>US KSU	Lr-0.14	Austrian Pietrain<br>Austrian crossbreeds<br>US KSU, Meishan
Lr-27.0	US KSU, PCV	Lr-0.15	Austrian Pietrain<br>Canadian Yorkshire<br>Meishan<br>US KSU, PCV, Big Pig

SLA class I haplotype	Population	SLA class II haplotype	Population
Lr-28.0	Austrian Pietrain<br>Canadian Landrace<br>Austrian crossbreeds<br>US PCV, Big Pig	Lr-0.19	Austrian Pietrain<br>Austrian crossbreeds<br>US KSU, PCV, Big Pig
Lr-29.0	Austrian Pietrain<br>Danish crossbreeds<br>US Big Pig	Lr-0.21	US KSU, PCV
Lr-32.0	Austrian Pietrain<br>Canadian Landrace<br>Austrian crossbreeds<br>Danish crossbreeds<br>US Big Pig	Lr-0.22	US PCV, Big Pig, Meishan
Lr-35.0	Austrian Pietrain<br>Canadian Landrace&Yorkshire<br>Austrian crossbreeds	Lr-0.23	Austrian Pietrain<br>Canadian Landrace&Yorkshire<br>Microminipig<br>Austrian crossbreeds<br>US Big Pig<br>Korean native pigs
Lr-37.0	Canadian Yorkshire	Lr-0.24	Austrian Pietrain<br>Canadian Yorkshire<br>US Big Pig
Lr-43.0	Austrian Pietrain<br>Austrian crossbreeds<br>US KSU	Lr-0.25	Austrian Pietrain<br>Austrian crossbreeds<br>US Big Pig
Lr-49.0	Danish crossbreeds	Lr-0.26	Austrian crossbreeds<br>US PCV, Big Pig
Lr-50.0	ST cell line	Lr-0.27	Canadian Yorkshire
Lr-57.0	Danish crossbreeds	Lr-0.36	ESK-4 cell line
Lr-58.0	Canadian Yorkshire		
Lr-63.0	Canadian Yorkshire		
Lr-70.0	Bama miniature pig		
Lr-76.0	Wuzhishan		
Lr-80.0	Bama miniature pig		
Lr-81.0	Bama miniature pig		
Lr-82.0	Bama miniature pig		

References: Austrian crossbreeds (50%LY/50%D) (Unpublished data, Hammer et al., 2015), Austrian Pietrain (Essler et al., 2013), Bama miniature pig (Gao et al., 2014), Canadian Landrace&Yorkshire (Gao et al., 2017), Microminipig (Ando et al., 2014), Danish crossbreeds (50%LY/50%D) (Pedersen et al., 2014), German Landrace (Gimsa et al., 2017), Korean native pigs (Cho et al., 2010), Meishan (Ho et al., 2006; Ho et al., 2010), US PCV, KSU (50%LY/50%D) Big Pig (50%L/50%Y) (Ho et al., 2009; Ho et al., 2010)

References

Ando, A., Imaeda, N., Ohshima, S., Miyamoto, A., Kaneko, N., Takasu, M., et al. (2014). Characterization of swine leukocyte antigen alleles and haplotypes on a novel miniature pig line, Microminipig. *Anim Genet* 45(6)**,** 791-798. doi: 10.1111/age.12199.

Cho, H.O., Ho, C.S., Lee, Y.J., Cho, I.C., Lee, S.S., Ko, M.S., et al. (2010). Establishment of a resource population of SLA haplotype-defined Korean native pigs. *Mol Cells* 29(5)**,** 493-499. doi: 10.1007/s10059-010-0061-8.

Essler, S.E., Ertl, W., Deutsch, J., Ruetgen, B.C., Groiss, S., Stadler, M., et al. (2013). Molecular characterization of swine leukocyte antigen gene diversity in purebred Pietrain pigs. *Anim Genet* 44(2)**,** 202-205. doi: 10.1111/j.1365-2052.2012.02375.x.

Gao, C., Jiang, Q., Guo, D., Liu, J., Han, L., and Qu, L. (2014). Characterization of swine leukocyte antigen (SLA) polymorphism by sequence-based and PCR-SSP methods in Chinese Bama miniature pigs. *Dev Comp Immunol* 45(1)**,** 87-96. doi: 10.1016/j.dci.2014.02.006.

Gao, C., Quan, J., Jiang, X., Li, C., Lu, X., and Chen, H. (2017). Swine Leukocyte Antigen Diversity in Canadian Specific Pathogen-Free Yorkshire and Landrace Pigs. *Front Immunol* 8**,** 282. doi: 10.3389/fimmu.2017.00282.

Gimsa, U., Ho, C.S., and Hammer, S.E. (2017). Preferred SLA class I/class II haplotype combinations in German Landrace pigs. *Immunogenetics* 69(1)**,** 39-47. doi: 10.1007/s00251-016-0946-6.

Hasegawa, M., Kishino, H., and Yano, T. (1985). Dating of the human-ape splitting by a molecular clock of mitochondrial DNA. *J Mol Evol* 22(2)**,** 160-174. doi: 10.1007/BF02101694.

Ho, C.S., Lunney, J.K., Franzo-Romain, M.H., Martens, G.W., Lee, Y.J., Lee, J.H., et al. (2009). Molecular characterization of swine leucocyte antigen class I genes in outbred pig populations. *Anim Genet* 40(4)**,** 468-478. doi: 10.1111/j.1365-2052.2009.01860.x.

Ho, C.S., Lunney, J.K., Lee, J.H., Franzo-Romain, M.H., Martens, G.W., Rowland, R.R., et al. (2010). Molecular characterization of swine leucocyte antigen class II genes in outbred pig populations. *Anim Genet* 41(4)**,** 428-432. doi: 10.1111/j.1365-2052.2010.02019.x.

Ho, C.S., Rochelle, E.S., Martens, G.W., Schook, L.B., and Smith, D.M. (2006). Characterization of swine leukocyte antigen polymorphism by sequence-based and PCR-SSP methods in Meishan pigs. *Immunogenetics* 58(11)**,** 873-882. doi: 10.1007/s00251-006-0145-y.

Kumar, S., Stecher, G., Li, M., Knyaz, C., and Tamura, K. (2018). MEGA X: Molecular Evolutionary Genetics Analysis across Computing Platforms. *Mol Biol Evol* 35(6)**,** 1547-1549. doi: 10.1093/molbev/msy096.

Pedersen, L.E., Jungersen, G., Sorensen, M.R., Ho, C.S., and Vadekaer, D.F. (2014). Swine Leukocyte Antigen (SLA) class I allele typing of Danish swine herds and identification of commonly occurring haplotypes using sequence specific low and high resolution primers. *Vet Immunol Immunopathol* 162(3-4)**,** 108-116. doi: 10.1016/j.vetimm.2014.10.007.

Smith, D.M., Martens, G.W., Ho, C.S., and Asbury, J.M. (2005). DNA sequence based typing of swine leukocyte antigens in Yucatan miniature pigs. *Xenotransplantation* 12(6)**,** 481-488. doi: 10.1111/j.1399-3089.2005.00252.x.
